# Fighting the invader: strategies against intracellular bacteria

**DOI:** 10.3389/fphar.2026.1904697

**Published:** 2026-08-20

**Authors:** Blanca Lorente-Torres, Pablo Castañera, Jesús Llano-Verdeja, Helena Á. Ferrero, Sergio Fernández-Martínez, Farzaneh Javadimarand, Luis M. Mateos, Álvaro Mourenza, Michal Letek

**Affiliations:** 1 Departamento de Biología Molecular, Instituto de Desarrollo Ganadero y Sanidad Animal (INDEGSAL), Universidad de León, León, Spain; 2 Instituto de Investigación Biosanitaria de León (IBIOLEÓN), Universidad de León, León, Spain; 3 Departamento de Biología Molecular, Instituto de Biología Molecular, Genómica y Proteómica (INBIOMIC), Universidad de León, León, Spain; 4 Instituto de Investigación Biomédica de A Coruña (INIBIC), A Coruña, Spain; 5 EXPRELA Group, Centro Interdisciplinar de Química e Bioloxía (CICA), Universidade da Coruña, A Coruña, Spain

**Keywords:** antimicrobial resistance, engineered anti-infective strategies, host–pathogen interplay, intracellular persistence, intracellular reservoirs

## Abstract

Intracellular pathogenic bacteria are often-overlooked agent of chronic, recurrent and difficult-to-treat infections in a context of escalating antimicrobial resistance. By hiding within professional and non-professional phagocytes, adopting dormant or slow-replicative states and rewiring host pathways, these pathogens successfully evade both immune clearance and conventional antibiotic therapy, thus contributing to treatment failure and, potentially, carcinogenesis. Here we synthesise how obligate and facultative intracellular bacteria invade and persist within host cells, and interpret these processes as interconnected, engineerable modules of the host-pathogen interplay. Using *Staphylococcus aureus* as a paradigm of facultative intracellular persistence across diverse tissues, we highlight how these pathways generate long-lived intracellular reservoirs that are poorly addressed by current drugs. Building on this framework, we discuss emerging strategies that explicitly apply engineering principles and tools to intracellular infections, including AI-assisted discovery of novel anti-infectives, rational design of host-directed therapies acting on host pathways, microbiota-based interventions, RNA-based approaches, drug repurposing and combinatorial regimens. We argue that integrating pathogen- and host-targeted strategies within a design-build-test-learn cycle, supported by computational modelling and careful attention to safety and pharmacokinetics, offers a route to engineer eradication of intracellular reservoirs, extend antibiotic lifespan and accelerate translation of new therapies against multidrug-resistant infections.

## Graphical Abstract

**Figure:**
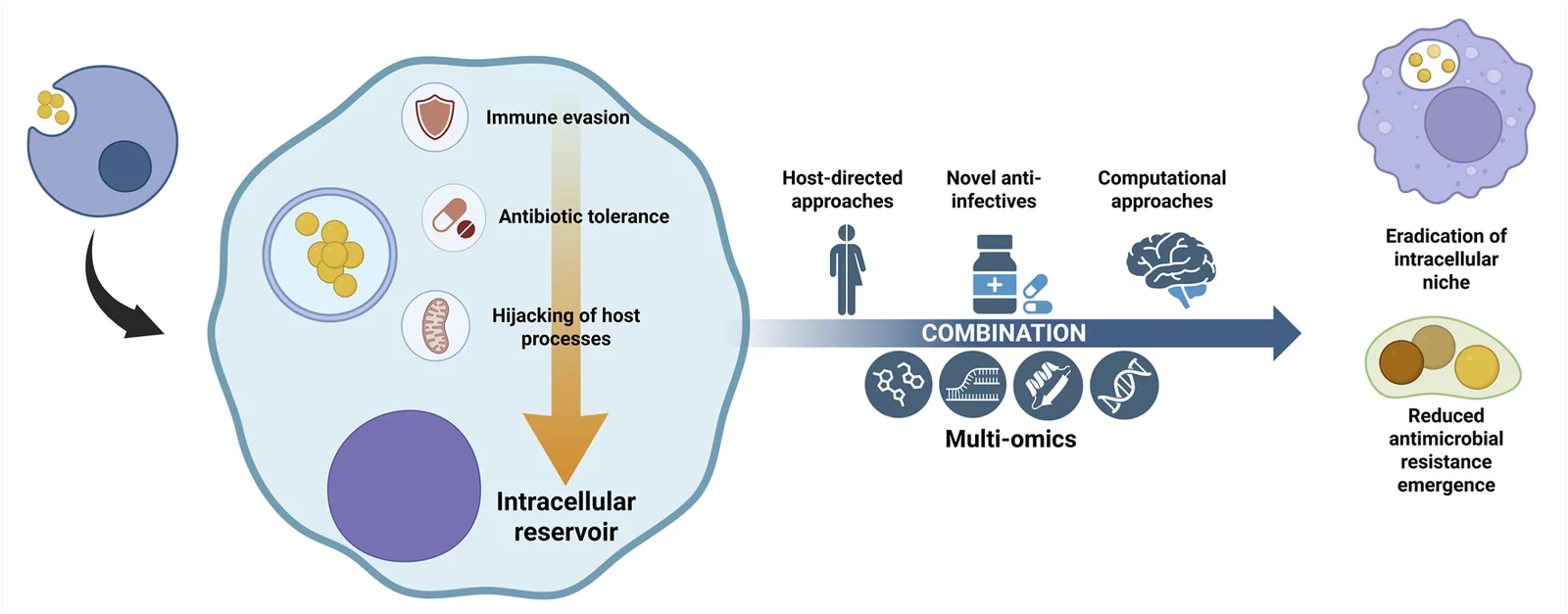
Intracellular bacterial pathogens can evade immune responses, tolerate antibiotic exposure, hijack host-cell processes, and establish persistent intracellular reservoirs. The proposed strategy integrates host-directed approaches, novel anti-infective compounds, computational tools, and multi-omics analyses to identify rational combination therapies. These combined interventions aim to disrupt the intracellular niche, improve eradication of intracellular bacteria, and reduce the emergence of antimicrobial resistance.

## 1 Introduction

The global emergence of antimicrobial resistance is turning bacterial infections into a major health challenge in the 21st century, now recognised as one of the leading causes of death worldwide (Vos et al., 2020). Only in 2019, 4.95 million deaths were associated with antibiotic-resistant bacteria, including 1.27 million deaths attributable to antibiotic-resistant bacteria, with a relevant prevalence of respiratory infections, claiming more than 1.5 million deaths. Leading the mortality rate board are the six bacteria belonging to ESKAPE, with methicillin-resistant *Staphylococcus aureus* (MRSA) becoming the leading cause of death in 135 countries (Murray et al., 2022; Naghavi et al., 2024).

In recent decades, few new antibiotic classes have been discovered, with limited clinical translation, and the lack of new effective treatments and the rapid development of resistance are slowly pushing us into the so-called “post-antibiotic era”. At the same time, an often-overlooked survival strategy aggravates this scenario, as certain bacterial pathogens adopt an intracellular lifestyle. Here, intracellular pathogens refer to bacteria that invade and persist within host cells as part of infection (Thakur et al., 2019). Related, but conceptually distinct, microbial communities may also inhabit tissues and influence tumour microenvironments, a context recently referred to as tumour microbiota (Fu et al., 2022; Schorr et al., 2023). The intracellular milieu offers protection from the immune system and from antibiotics, which must cross subcellular compartments and even withstand acidic conditions, commonly resulting in reduced efficacy (Kember et al., 2022; Uribe-Querol and Rosales, 2017). In addition, some of these pathogens can enter a slow or non-replicative state that favours antibiotic tolerance (Watson et al., 2020). Therefore, treatment failure leads to the emergence of chronic infections caused by intracellular persisters, which lead to inadequate clearance (Peyrusson et al., 2020). Many antibiotics show reduced efficacy against intracellular bacteria *in vitro*, with important therapeutic implications. This limitation is particularly relevant given the growing evidence linking intracellular bacteria to chronic infection and, in some contexts, cancer (Fu et al., 2022; Schorr et al., 2023; Kamaruzzaman et al., 2017). Should this trend persist, the therapeutic options to face antibiotic resistant infections. Therefore, there is an urgent need to change the therapeutic landscape to allow access to new perspectives and innovative strategies. In this review, we discuss how the intracellular lifestyle of multidrug-resistant pathogens reshapes the therapeutic landscape and highlight emerging pharmacological strategies that target both bacteria and the host.

## 2 Intracellular lifestyle and clinical reservoirs of multidrug-resistant bacteria

Intracellular lifestyles likely emerged from trophic interactions between early eukaryotes and bacteria, in which ingested bacteria that evaded digestion could become either parasitic or symbiotic. Thus, the appearance of intracellular pathogenesis may be intrinsically linked to predation and endosymbiotic existence (Casadevall, 2008; Martyn et al., 2022). Intracellular bacteria can be classified as obligate or facultative. Obligate bacteria, such as *Rickettsia* spp. and *Chlamydia* spp., have lost the capacity to survive outside host cells, while facultative bacteria retain the ability to persist in the extracellular environment. The intracellular niche provides protection, nutrients and, in some contexts, a vehicle for dissemination, and can be considered a relatively constant and predictable environment to adapt to, although further transmission often requires host tissue damage or host-to-host transfer mechanisms (Casadevall, 2008).

Several pathogenic bacteria follow this strategy. *Mycobacterium tuberculosis*, for example, is a facultative intracellular pathogen and the causative agent of one of the deadliest infectious diseases in human history, which still prevails today (Behr et al., 2018; World Health Organization, 2023). *Listeria monocytogenes* is another example, as an opportunistic, facultative intracellular pathogen associated with increased mortality in immunocompromised patients, the elderly, and pregnant women (McLauchlin et al., 2004; Lungu et al., 2011; Buchanan et al., 2017). Over the years, accumulating evidence has revealed that many bacterial species once considered strictly extracellular can, in fact, adopt intracellular lifestyles. Beyond classical intracellular pathogens such as *Salmonella* spp., *Rickettsia* spp., *Shigella* spp. and *Pseudomonas* spp. (Stern et al., 1985; Sansonetti et al., 1986; Ricketts, 1991; Richter-Dahlfors et al., 1997), the broader recognition that many bacteria can adopt intracellular lifestyles has further expanded our understanding of host–microbe interactions (Fu et al., 2022). This perspective has shed light on how intracellular colonization can shape pathogenesis and even contribute to chronic diseases. This concept is illustrated by *Helicobacter pylori*, a well-established risk factor for gastric cancer (Cover, 2016). Despite this, intracellular phases have often been largely overlooked. Throughout this review, we distinguish intracellular reservoirs from broader tissue-associated bacterial detection: the term reservoir is used when intracellular localization, persistence and potential relevance to treatment failure are supported, whereas tissue or tumour detection alone is treated more cautiously as evidence of association.

A good example is *Staphylococcus aureus*, for which a growing body of evidence supports its intracellular nature, although it has traditionally been regarded as an extracellular bacterium. Despite being discovered in the 19th century, observations of its intracellular survival were not reported until 1952, and later within non-professional phagocytes *in vitro* (Rogers and Tompsett, 1952; Hamill et al., 1986). *S. aureus* is a facultative intracellular bacterium and a common commensal, with nasal carriage increasing the risk of invasive infection (Foster, 2004; Brown et al., 2014). Its intracellular lifestyle has been widely demonstrated *in vitro*, in epithelial and endothelial cells, osteoclasts, keratinocytes, fibroblasts, macrophages and neutrophils (Rogers and Tompsett, 1952; Bayles et al., 1998; Gresham et al., 2000; Menzies and Kourteva, 2000; Nair et al., 2003; Koziel et al., 2009). Notably, intracellular *S. aureus* has also been documented within cells from tissues dissected from infected patients. Clinical isolates from difficult-to-treat *S. aureus* infections frequently show persister phenotypes such as small-colony variants (Bär et al., 2022; Huemer et al., 2021). Host-mediated stress conditions promote the development of persisters, characterized by downregulated virulence, slow replication and upregulation of ribosomal and metabolic pathways (Huemer et al., 2021; Wong Fok Lung et al., 2019). Genetically identical isolates recovered from methicillin-susceptible *S. aureus* (MSSA) infections despite prolonged antibiotic therapy support the idea that phenotypic adaptation is driven by the host environment rather than stable genetic change (Huemer et al., 2021).

Although the nasal vestibule has traditionally been considered the main reservoir of *S. aureus*, recent clinical work suggests that the broader nasal cavity is also an important site of colonization, with intracellular carriage detected in 5.6% of patients, whereas overall carriage at the vestibule and broader nasal-cavity sites was 32.2%–33.3% (Rigaill et al., 2023). In the respiratory tract, *S. aureus* cocci have been observed within macrophages from cystic fibrosis patients, forming presumed reservoirs for chronic pneumonia (Li et al., 2017). Cutaneous tissues also represent a niche for intracellular *S. aureus*, with bacteria internalizing skin keratinocytes and evading antibiotic killing, and strategies such as hyaluronan-based nanogels have been explored to target intracellular bacteria in persistent skin infections (Al et al., 2019; Montanari et al., 2020). Intracellular infection by *S. aureus* is particularly well established in osteomyelitis (Hamza et al., 2013; Zelmer et al., 2022). Small-colony variants are especially implicated in prosthetic bone infections, contributing to relapsing or treatment-refractory cases (Sendi et al., 2006). In bone and joint infections, *S. aureus* can persist within musculoskeletal cells, promoting recurrence; the resulting intracellular pro-inflammatory state can degrade both bone and cartilage (Zelmer et al., 2022; Alder et al., 2020). Clinical confirmation of *S. aureus* infection within osteocytes further supports the role as reservoirs for persistent infections (Yang et al., 2018). Recent studies using AI-assisted analysis of biopsies from patients with *S. aureus* infection have revealed that most bacteria reside within non-classical monocytes or macrophages, with cell death and the expulsion of extracellular traps (ETosis) serving as mechanisms of bacterial release (Monteith et al., 2021; von Köckritz-Blickwede and Winstel, 2022; Tripathi et al., 2025). Interestingly, recent research suggests that some human cancers may harbour low-biomass microbial ecosystems, including intracellular bacteria, with *S. aureus* signatures detected in melanoma, breast and lung cancer tissues (Fu et al., 2022; Schorr et al., 2023). These findings broaden the clinical implications of *S. aureus* intracellular persistence and its potential role across diverse human pathologies, and challenge the commonly accepted view of *S. aureus* as a primarily extracellular pathogen (Fu et al., 2022; Schorr et al., 2023). In turn, this illustrates how simplistic current concepts of antibiotic therapy can be and raises the question of whether other bacteria occupy overlooked intracellular niches in health and disease.

## 3 The silent intruder: how intracellular pathogens infiltrate and exploit host cells

### 3.1 Early innate sensing and antimicrobial defences

The outcome of infection depends on the dynamic interplay between host and pathogen. To successfully establish disease, microbes must breach anatomical barriers, evade local defences, replicate within host cells and tissues, disseminate within the host, and suppress or subvert immune responses (Syed et al., 2024). Survival in this context relies heavily on active communication, such as quorum sensing, which enables bacteria to coordinate behaviours including biofilm formation, virulence factor expression, secondary metabolite production and stress adaptation, often *via* specialised secretion systems (Pena et al., 2019). Secretion systems have evolved as a means to withstand stressful conditions, including interspecies competition, and at least eight types have been described with functions ranging from environmental adaptation and niche establishment to protein translocation across bacterial membrane or delivery into host cells, all contributing to virulence and pathogenesis (Pena et al., 2019). Several secretion systems (types I–VII) are directly implicated in immune evasion and thus form a central component of the bacterial virulence arsenal (Escoll, 2017).

Early control of infection relies on rapid sensing of conserved microbial and stress signals by pattern-recognition receptors (PRRs), including Toll-like receptors (TLRs), C-type lectin receptors, NOD-like receptors and RIG-I-like receptors, which detect pathogen- and damage-associated molecular patterns (PAMPs; DAMPs) and trigger inflammatory and antimicrobial programmes (Thakur et al., 2019; Schenten and Medzhitov, 2011). Alongside these receptors, cGAS/STING pathway senses cytoplasmic DNA, either from microbial or host-damage origin, and activates cGAS (cyclic guanosine adenosine synthase), activating key signalling hubs such as TBK1, NF-κB and MAPKs, leading to type I interferon production, cytokine induction and, in some contexts, programmed cell death (Hopfner and Hornung, 2020; Maculins et al., 2016; Mukherjee and Dikic, 2022; Tong et al., 2024). On the other hand, TLRs recognize a broad range of PAMPs, with signalling mainly proceeding *via* MyD88, rapidly inducing cytokines such as TNF-α, IL-1β, IL-6 and IL-8 that orchestrate early antimicrobial responses, while many pathogens counter this detection by modifying surface ligands, undergoing antigenic variation or actively suppressing TLR-driven cytokine production (Erridge et al., 2002; Christie et al., 2005; Obergfell and Seifert, 2015; DePaolo et al., 2008; Nakayama et al., 2012).

Endothelial cells rapidly respond to infection by upregulating trafficking and adhesion molecules upon sensing cytokines, eicosanoids, and microbial products (Butcher, 1991; Tosi, 2005). This leads to increased expression of P-selectin and E-selectin on the endothelium, enabling the characteristic “slow rolling” behaviour of neutrophils and monocytes prior to ICAM-1 binding and activation. Once activated, microbial uptake—phagocytosis—and oxygen-dependent (producing reactive oxygen species; ROS) and oxygen-independent killing take place (Tosi, 2005; Ley, 2002). Among professional phagocytes, macrophages and neutrophils are found, while non-professional phagocytes include epithelial cells and keratinocytes. Uptake begins at the plasma membrane, where receptors initiate internalization either through direct PRR recognition or opsonization, triggering the formation of a phagosome, which matures into a phagolysosome enriched in ROS, reactive nitrogen species (RNSs), proteases, and other antimicrobial factors (Thakur et al., 2019; Flannagan et al., 2012; Levin et al., 2016).

Macrophages are central sentinels of immune surveillance, clearing microbes by phagocytosis and initiating adaptive responses through antigen presentation (Syed et al., 2024; Westman and Grinstein, 2021). They are often simplistically classified into pro-inflammatory, microbicidal M1-like states driven by Th1 stimuli (IFN-γ, LPS, TNF-α) and tissue-repairing M2-like states associated with Th2 cytokines (IL-4, IL-10, TGF-β), which dampen nitric oxide production and promote fibrosis and tissue remodelling (Mills et al., 2016; Huang et al., 2014). Several intracellular pathogens, including *Listeria monocytogenes*, *Salmonella enterica* and *M. tuberculosis*, reprogram macrophage polarisation to create niches that limit tissue damage yet favour long-term bacterial persistence (Biswas et al., 2007; Kahnert et al., 2006; Huang et al., 2015). In parallel, efferocytosis—the clearance of apoptotic infected cells—normally restricts infection and inflammation but can be hijacked by pathogens such as *L. monocytogenes* to promote cell-to-cell spread and immune evasion; importantly, non-professional phagocytes such as epithelial and endothelial cells also participate in apoptotic cell clearance and help limit inflammatory spread (Nagata, 2018; Czuczman et al., 2014; Behar and Briken, 2019; Sihombing et al., 2021).

During infection, macrophages undergo rapid metabolic rewiring, shifting from predominantly oxidative metabolism toward aerobic glycolysis, with citrate and other tricarboxylic acid (TCA) cycle intermediates supporting inflammatory programmes under type I IFN and NF-κB control (Hsieh et al., 2020; Jha et al., 2015; Bhagwat et al., 2025). Key immunometabolites such as itaconate and succinate exert direct antibacterial pressure and stabilise HIF-1α, yet several intracellular pathogens can degrade or bypass these constraints, while host-derived citrate can even modulate *S. aureus* carbon metabolism and attenuate virulence (Tannahill et al., 2013; Chen et al., 2020; Chen et al., 2024). Together, these immunometabolic circuits shape trained immunity and persistence, and intersect with cytoskeletal and organelle remodelling in ways that influence whether infection is contained or progresses to chronic intracellular colonisation (Mukherjee and Dikic, 2022; Palma Medina et al., 2020).

### 3.2 Host cell invasion

Successful colonisation begins with attachment to the extracellular matrix or mucosal surfaces *via* bacterial adhesins, which shape bacterial tropism for the host and tissues (Barber and Fitzgerald, 2024). Beyond classical phagocytosis, many intracellular pathogens exploit lipid-raft/caveolin-mediated endocytosis or exocytosis-coupled uptake in non-professional phagocytes, where caveolae and associated regulators route microbes to less degradative compartments and modulate signalling to favour persistence (Barman and Drolia, 2024). *S. aureus* uses a zipper-like process driven by fibronectin-binding proteins (FnBPs) engaging α5β1 integrins, with wall teichoic acids and clumping factor B reinforcing nasal adhesion, while raft integrity and α-haemolysin further promote internalisation into epithelial and endothelial cells (Weidenmaier et al., 2008; Sinha and Fraunholz, 2010; Fraunholz and Sinha, 2012; Goldmann et al., 2024). *L. monocytogenes* coordinates multiple routes in non-phagocytic cells, notably InlB-Met and InlA-E-cadherin pathways coupled to LAP-Hsp60-Cav-1 signalling, which loosen tight junctions, recruit caveolin and PI3K, and drive transcytosis across the intestinal barrier (Barman and Drolia, 2024; Ireton et al., 2018; Radoshevich and Cossart, 2018; Drolia and Bhunia, 2019). Likewise, *M. tuberculosis* engages raft-localised receptors on myeloid cells, inducing caveolin-dependent invagination and dynamin-mediated vesicle scission, thereby avoiding phagolysosomal fusion and sustaining intracellular survival (Barman and Drolia, 2024; Kotzé et al., 2020).

Beyond manipulating uptake and actin dynamics, intracellular pathogens must also withstand host-derived oxidative stress. Low-molecular-weight thiols such as glutathione, mycothiol and bacillithiol are central redox buffers that support bacterial fitness and survival under oxidative conditions by maintaining protein function and enabling detoxification pathways, which is especially relevant in the context of infection (Ku and Gan, 2021). Increasing evidence indicates that these molecules also modulate virulence in several intracellular pathogens, including *L. monocytogenes*, *M. tuberculosis* and *S. aureus*, as mutants defective in thiol biosynthesis or recycling are attenuated and display reduced fitness (Ku and Gan, 2021; Mehta et al., 2016; Mikheyeva et al., 2019; Posada et al., 2014; Reniere et al., 2015). In *S. aureus*, bacillithiol and its associated bacilliredoxin pathway enhance survival within host cells by defending against oxidative stress, while in *M. tuberculosis* phagosomal acidification perturbs the mycothiol redox potential, which in turn influences virulence gene expression (Mehta et al., 2016; Mikheyeva et al., 2019). Moreover, host glutathione can act as a signal that boosts intracellular glutathione levels in *L. monocytogenes* and activates virulence programmes, illustrating how host and bacterial thiol pools intersect to shape intracellular infection outcomes (Reniere et al., 2015).

Following receptor engagement, uptake relies on tight spatial and temporal control of host actin dynamics, coordinated by Rho GTPases, ubiquitin-dependent regulation and exocytosis-coupled remodelling (Mukherjee and Dikic, 2022). Many pathogens manipulate these pathways to induce membrane ruffles, stabilise pathogen-containing vacuoles or promote direct cytosolic access; for example, *S. enterica* uses T3SS-1 effectors SopE and SptP to drive Cdc42-dependent actin rearrangements that are sensed by the host and activate NF-κB and inflammasomes (Mukherjee and Dikic, 2022). In *S. aureus*, FnBP-integrin interactions trigger local Cdc42/N-WASP/Arp2/3-mediated actin polymerisation at focal adhesions, followed by exocytic delivery of Cdc42GAP to terminate signalling and allow phagocytic cup sealing and vesicle closure (Fraunholz and Sinha, 2012; Schröder et al., 2006; Rauch et al., 2016). *L. monocytogenes* similarly couples InlB–Met entry to dynamin-2 recruitment and, after phagosomal escape, expresses ActA to hijack Arp2/3 and generate actin “comet tails” that drive intracellular motility and direct cell-to-cell spread, a strategy shared with *Shigella flexneri* and spotted-fever group *Rickettsia* spp. (Barman and Drolia, 2024; Lambrechts et al., 2008; Sit and Lamason, 2024).

### 3.3 Phagosomal escape and autophagy

Intracellular pathogenic bacteria can be broadly divided into vacuolar pathogens, which remain within membrane-bound compartments, and cytosolic pathogens, which escape into the host cytoplasm and are subject to distinct sensing and clearance mechanisms. Once internalised, pathogen-containing phagosomes normally undergo a maturation process in which sequential fusion with endosomes and lysosomes delivers V-ATPases, NADPH oxidase, antimicrobial peptides and hydrolytic enzymes, leading to lumen acidification, production of ROS and RNS, as well as metal depletion, ultimately killing engulfed bacteria (Flannagan et al., 2012; Westman and Grinstein, 2021; Buckley et al., 2016; Guan and Liu, 2020). To withstand these hostile conditions, vacuolar intracellular bacteria have evolved convergent strategies that include arresting phagosome-lysosome fusion or even excluding V-ATPases, as happens with *M. tuberculosis* and *S. enterica*, which remodel phosphoinositides and endosomal trafficking *via* phosphatases and T3SS effectors respectively (Hernandez et al., 2004; Campoy and Colombo, 2009; Augenstreich et al., 2019; Nasiri and Venketaraman, 2025). Other bacteria turn the vacuole into a specialised niche, as *Legionella pneumophil*a or *Coxiella burnetii* (Westman and Grinstein, 2021; Campoy and Colombo, 2009). Another strategy is to neutralize acidity through ammonia production, such as *H. pylori* and *S. aureus* (Volk et al., 2024; Weinrick et al., 2004; Schwartz and Allen, 2006). Bacteria can also escape physically to the cytosol, as is the case of *L. monocytogenes* and *S. flexneri*, which deploy pore-forming toxins and phospholipases to rupture the phagosomal membrane and then exploit actin-based motility for intracellular spread, while *S. aureus*, for instance, employs Agr/Sar-regulated toxins and phenol-soluble modulins to damage phagosomal membranes (Campoy and Colombo, 2009; Volk et al., 2024; Baxt and Goldberg, 2014; Wozniak et al., 2020).

Ubiquitination and the ESCRT machinery shape vesicle trafficking, membrane repair and autophagy, and are therefore central to intracellular host defence, but many pathogens deploy E3 ligases, deubiquitinases and ERAD-hijacking toxins to remodel these pathways, build replication-permissive vacuoles and blunt antigen presentation (Maculins et al., 2016; Ribet and Cossart, 2018; Hurley, 2015; Rivera-Cuevas and Carruthers, 2023). Autophagy represents an additional barrier, whereby ULK1 activation, mTORC1 inhibition and PI3P-dependent recruitment of ATG proteins drive LC3 lipidation and adaptor-mediated recognition of ubiquitinated bacterial or vacuolar surfaces, promoting their xenophagic delivery to degradative autolysosomes (Jung et al., 2009; Franco et al., 2017; Nair et al., 2010). Intracellular pathogens such as *M. tuberculosis*, *S. enterica*, *S. flexneri*, *L. monocytogenes*, *C. burnetii*, *L. pneumophila* and *S. aureus* differentially expose, conceal or reshape damaged vacuoles, and by hijacking ubiquitin, ESCRT and autophagy pathways they tune xenophagic recognition and repair, often establishing LC3-positive, leaky or acidic niches where delayed lysosomal fusion and rewired host metabolism can paradoxically support bacterial replication (Nasiri and Venketaraman, 2025; Missiakas and Winstel, 2021; Bravo-Santano et al., 2018).

### 3.4 Programmed cell death

Programmed cell death shapes cellular homeostasis, embryonic development and tissue regeneration, and can be triggered by a wide range of signals, including cellular stress (Missiakas and Winstel, 2021). Accordingly, programmed cell death plays an important role during infection, generally favouring the host and harming invading pathogens (Behar and Briken, 2019). Programmed cell death comprises both non-inflammatory apoptosis and lytic, highly inflammatory processes such as necroptosis, pyroptosis and ferroptosis, all of which shape antimicrobial defence and tissue injury and can either restrict or promote pathogen dissemination depending on timing and tissue context (Behar and Briken, 2019; Soe et al., 2021).

To clear infection, phagocytes also engage efferocytosis, a key innate mechanism by which apoptotic infected cells are removed, thereby limiting both pathogen burden and inflammation (Nagata, 2018; Behar and Briken, 2019). This process is not restricted to professional phagocytes; epithelial and other non-professional phagocytes also contribute to apoptotic cell clearance (Sihombing et al., 2021). However, some pathogens subvert efferocytosis. For example, *L. monocytogenes* disrupts phagosomal membranes by secreting listeriolysin O (LLO) and then exploits efferocytosis of infected apoptotic cells to promote dissemination and avoid extracellular killing, while others use efferocytosis to dampen macrophage activation or actively avoid this route altogether (Czuczman et al., 2014; Behar and Briken, 2019; Dalboni et al., 2021).

Many obligate (*Chlamydia trachomatis* and *C. burnetii*) and facultative intracellular bacteria (*M. tuberculosis*, *L. pneumophila* and *S. enterica*), suppress apoptosis early during infection by increasing anti-apoptotic Bcl-2 family members, blocking Bax/Bak activation or neutralising TNF signalling to preserve their intracellular niche, and later redirect death pathways toward necrosis, necroptosis or pyroptosis *via* TNFR1-RIPK1-RIPK3 or inflammasome-gasdermin D signalling to facilitate escape and spread (Behar and Briken, 2019; Barber and Fitzgerald, 2024; Balcewicz-Sablinska et al., 1998; Chowdhury et al., 2020; Walsh et al., 2022). Some bacteria are more plastic, such as *S. aureus,* which employs toxins and regulators such as α-toxin (Hla), leukocidins, PSMs and other virulence factors to induce or delay apoptosis, trigger necroptosis and multiple inflammasomes, or divert inflammasome platforms into autophagic degradation, thereby disabling phagocytes, reshaping local inflammation and supporting long-term intracellular persistence and tissue damage (Fraunholz and Sinha, 2012; Missiakas and Winstel, 2021; Soe et al., 2021). By lowering Agr/SaeR-driven toxin output and increasing Bcl-2 and Mcl-1 levels, *S. aureus* reduces cytotoxicity and prolongs intracellular survival (Soe et al., 2021; Kubica et al., 2008; Sause et al., 2017). Alternatively, T7SS effectors such as EsxA directly interfere with epithelial apoptosis and, more broadly, T7SS dampens pro-inflammatory cytokine production in dendritic cells, allowing *S. aureus* to disable host cells without eliciting strong inflammatory responses (Soe et al., 2021; Korea et al., 2014; Warne et al., 2016; Ohr et al., 2017; Ulhuq et al., 2020).

### 3.5 Nutritional immunity and metabolic hijacking

Intracellular infection establishes a metabolic tug-of-war in which host cells deploy nutritional immunity while pathogens evolve several countermeasures to secure nutrients (Bhagwat et al., 2025; Escoll and Buchrieser, 2018; Traven and Naderer, 2019). The host restricts access to metals such as iron, zinc, manganese and copper, and uses the TCA-derived metabolites and glycolytic rewiring as antimicrobial tools (Ezzeddine and Ghssein, 2023). Meanwhile, a Warburg-like shift with citrate-to-itaconate and succinate accumulation activates Nrf2, stabilises HIF-1α, drives IL-1β, and fuels fatty acid and prostaglandin synthesis (Bhagwat et al., 2025; Tannahill et al., 2013; Michelucci et al., 2013). In parallel, control of amino acids such as arginine, tryptophan, asparagine and glutamine shapes effector responses and constrains pathogen growth (Bhagwat et al., 2025; Olive and Sassetti, 2016).

However, pathogens subvert these pressures by developing high-affinity metal uptake and flexible carbon use, and by actively reshaping host metabolism. Metallophores and specific transport systems, as is the case of HupDGC and Fhu in *L. monocytogenes*, multiple siderophores in *S. enterica* serovar Typhimurium, and Mn/Zn transporters in *M. tuberculosis*, enable metal scavenging despite host sequestration (Parrow et al., 2013; Choudhury et al., 2021; Mohsen et al., 2023). *Mycobacterium tuberculosis* relies on host lipids and fatty acids, inducing lipid droplet biogenesis *via* urease C, perturbing DNA repair and activating cGAS/STING, while suppressing autophagy and establishing a lipid-rich, immunosuppressed niche (Stutz et al., 2018; Anes et al., 2023; Passos et al., 2024). *S.* Typhimurium favours aerobic glycolysis over a complete TCA cycle to promote inflammation and host metabolic reprogramming (Wang et al., 2021). In contrast, *S. aureus* shifts to Rex-regulated anaerobic metabolism and uses its glycolysis to activate host HIF-1α, driving host glycolysis, necroptosis and starvation-induced autophagy that increase bacterial uptake, deplete glucose and amino acid pools, and enhance glutamine catabolism (Wong Fok Lung et al., 2019; Volk et al., 2024; Bravo-Santano et al., 2018; Bravo-Santano et al., 2019a). Some host fatty acids, such as docosanoic, icosanoic and palmitic acid, increase upon *S. aureus* infection, acting in response to infection with cytoprotective roles (Bravo-Santano et al., 2019a; Quehenberger et al., 2011).

Mitochondria act as central hubs, with infection extensively remodelling mitochondrial dynamics, respiration and quality control (Escoll, 2017; Mukherjee and Dikic, 2022; Escoll et al., 2021). Many bacteria perturb fission-fusion balance and mitophagy, diverting E3 ligases, deubiquitinases, LC3, LIR-containing receptors and PARKIN from organelle surveillance toward xenophagy or toward pathways that favour intracellular survival (Mukherjee and Dikic, 2022; Manzanillo et al., 2013). Pore-forming toxins such as VacA, in H. pylori, and LLO, in *L. monocytogenes*, for example, increase mitochondrial outer membrane permeabilization, promoting cytochrome c release, ROS and mitochondrial DNA leakage that ultimately activate inflammasomes and TLR-linked inflammatory cascades, while other bacterial effectors subtly modulate mitochondrial outer-membrane permeabilization and the ubiquitination of inflammasome components, dampening apoptosis or caspase-1-driven pyroptotic cell death to evade inflammatory clearance (Mukherjee and Dikic, 2022; Galmiche and Rassow, 2010; Juliana et al., 2012).

### 3.6 Genotoxic stress in infection

Beyond contributing to genomic instability, infection-associated DNA damage can reshape host cell fate, chromatin state and checkpoint control in ways that favor intracellular persistence and expose host-directed vulnerabilities in DNA repair and epigenetic regulation. Upon infection, one of the most surprising consequences is that pathogens can compromise the stability of the host genome. This damage triggers the DNA damage response (DDR), a conserved signalling network that preserves genome integrity (Papamichos-Chronakis and Peterson, 2013; Wu et al., 2024). DDR and checkpoint pathways not only focus on DNA repair but also determine cell fate: when damage exceeds the capacity of recovery, DDR triggers either senescence or death (Zhao et al., 2023; Jeon et al., 2024). Consequently, failure of the DDR machinery results in the accumulation of DNA damage and genomic instability, thereby contributing to disease development (Kwok et al., 2024; Siametis et al., 2024; Berkova et al., 2025).

An increasing body of evidence indicates that bacterial infections can induce DNA damage in host cells (Hopfner and Hornung, 2020; Imai et al., 2021). This damage may arise directly from toxins and other genotoxic factors, or indirectly *via* alterations of immune defence mechanisms (Chumduri et al., 2016; Bednarski and Sleckman, 2019). DNA damage can be classified as base modifications (e.g., alkylation, mispaired bases), intrastrand and interstrand crosslinks, single-stranded breaks, and double-stranded breaks (DSBs) (Tiwari and Wilson, 2019). DSBs are less common and may come from the convergence of two single-stranded breaks or other primary lesions, but they occur more frequently during infection due to hijacking by pathogens of pivotal cellular signalling pathways that generate DNA damage (Tong et al., 2024; Berkova et al., 2025).

Part of this damage comes from persistent activation of TLR2, causing replication stress and DNA damage in macrophages, inducing pro-inflammatory responses to promote clearance (Berkova et al., 2025; Herrtwich et al., 2016; Morgan et al., 2022). ROS and RNS serve as signalling molecules, yet their accumulation drives oxidative stress and the formation of DNA lesions, including single-stranded breaks and DSBs (Fang, 2004; Trachootham et al., 2009). Alterations in DDR signalling pathways can generate mutations, promote chronic inflammation, impair immune function, and contribute to cancer development (Berkova et al., 2025; Groelly et al., 2023). Epigenetic reprogramming is tightly linked to DNA damage: extensive or persistent damage may overwhelm repair and is associated with changes such as DNA methylation and histone modifications (Wu et al., 2024). Bacterial damage can induce epigenetic modifications, such as aberrant DNA methylation or histone post-translational modifications (Berkova et al., 2025; Millán-Zambrano et al., 2022; Wu et al., 2023). *In vivo* studies comparing germ-free and conventional mice show reduced epithelial DNA methylation in germ-free animals, which underscores the role of microbes in shaping host epigenetic landscapes (Heppert et al., 2021). These changes are often driven by bacterial toxins and virulence factors (Berkova et al., 2025; Hamon et al., 2007; Lebreton et al., 2011).

Bacteria actively exploit DDR and nuclear signalling to enhance survival, replication and persistence (Hopfner and Hornung, 2020; Berkova et al., 2025; Chumduri et al., 2016). Many pathogens secrete nucleomodulins and cyclomodulins that enter the nucleus, mimic or inhibit host DNA methyltransferases (DNMTs) and chromatin regulators, modifying histones, and alter cell-cycle checkpoints, thereby reprogramming immune-gene expression, compromising barrier integrity and, in some cases, even promoting genomic instability and tumorigenesis (Berkova et al., 2025; Nougayrède et al., 2005; Escoll et al., 2016). Additional genotoxins and metabolites directly induce DNA breaks, oxidative lesions and chromosomal alterations, feeding into chronic inflammation and cancer-prone lesions (Berkova et al., 2025; Ding et al., 2019; Wong and Yu, 2019; Okita et al., 2020; Poncin et al., 2019).

*Staphylococcus aureus* is emerging as an example of how an intracellular pathogen hampers this network. It induces ROS-mediated DNA lesions, including 8-oxoguanine that can convert into DSBs, and accumulates γH2AX foci independently of classical apoptosis pathways (Berkova et al., 2025; Deplanche et al., 2019; Deplanche et al., 2015). In parallel, *S. aureus* reshapes the host epigenome *via* lactate-mediated HDAC11 inhibition and enhanced IL-10, Hla-driven rewiring of Th17 chromatin, altered DNA methylation and histone marks in airway epithelia, and downregulation of DNMTs and NuRD/PRC1 components in osteoblasts, collectively favouring an anti-inflammatory, pro-persistence state (Berkova et al., 2025; Modak et al., 2014; Pérez-Novo and Bachert, 2015; Heim et al., 2020; Nicolas et al., 2022; Howden et al., 2023). *S. aureus* delays G2/M and concentrates intracellular replication in G2, a phase with reduced antimicrobial peptide expression, thereby coupling cell-cycle stage to chromatin remodelling, DDR efficiency and intracellular survival (Berkova et al., 2025; Deplanche et al., 2015; Alekseeva et al., 2013).

**FIGURE 1:**
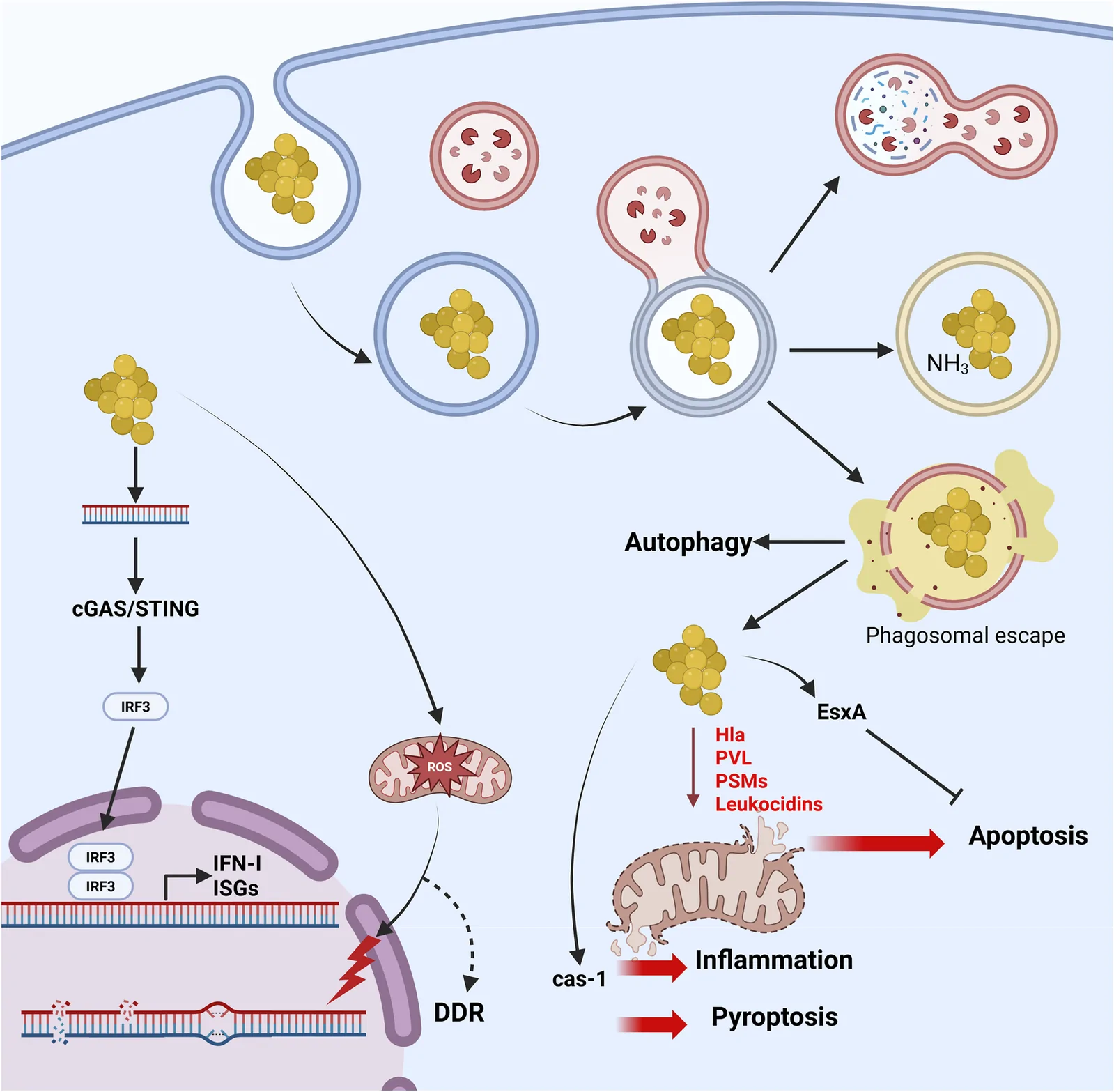
Intracellular *S. aureus* outcomes. Following uptake, *S. aureus*-containing vacuoles can undergo endosomal maturation towards lysosomal degradation. However, *S. aureus* can promote its survival within the endosome by neutralising the pH *via* ammonia (NH3) production or by escaping the phagosome into the cytosol. Bacterial effectors (e.g., EsxA) and toxins (Hla, PVL, PSMs, leukocidins) promote phagosomal rupture, mitochondrial damage, and ROS generation, leading to apoptosis, inflammation, and caspase-1-dependent pyroptosis. Moreover, the presence of cytosolic bacterial DNA triggers the cGAS/STING-IRF3 axis, including type I interferon (IFN-I) and interferon-stimulated genes (ISGs), thereby orchestrating antimicrobial defences and enhancing inflammation. Additionally, DNA damage induced by bacterial presence (*e.g.*, host oxidative burst) initiates DDR, which further shapes host defence and pathology.

Taken together, infection-associated genotoxic stress links intracellular persistence to host-cell fate decisions, chronic inflammation, ROS/RNS exposure, DDR activation and epigenetic rewiring. These processes are relevant to intracellular infection not because carcinogenesis is a separate focus of this review, but because they reveal host pathways that bacteria can exploit and may also represent host-directed therapeutic targets. Figure 1 provides an overview of the host–pathogen interactions discussed above, using intracellular *S. aureus* as an illustrative example.

## 4 Therapeutic landscape: new approaches

From their introduction in the 20th century, antibiotics have been used not only in human health but also in agriculture, livestock and fish farming, as treatments, prophylactics and growth promoters (Kaur Sodhi and Singh, 2022). Mechanistically, their mode of action includes inhibition of DNA replication, including through blockade of folate biosynthesis pathways, and inhibition of protein and cell-wall synthesis (Fàbrega et al., 2009; Syroegin et al., 2022). However, considering host-pathogen interplay reveals an additional array of targets, largely located in the infected cell rather than in the bacterium itself. Intracellular bacteria depend on, and systematically remodel, host autophagy and ESCRT machinery, mitochondrial dynamics, immunometabolism, apoptosis and DDR, suggesting that these pathways can be pharmacologically redirected to favour bacterial clearance instead of persistence (Pena et al., 2019; Mukherjee and Dikic, 2022; Campoy and Colombo, 2009; Berkova et al., 2025). For example, recent work showed that a non-canonical autophagic burst accompanying apoptosis can sequester intracellular ATP, which ultimately promotes immune silence (Terraza-Silvestre et al., 2025). Dissecting how intracellular infections exploit these host processes therefore opens new mechanistic targets and therapeutic strategies to tackle bacterial disease. A comparative overview is provided in Table 1.

**TABLE 1:** Comparison of pharmacological strategies for intracellular bacterial infections.

Strategy		Advantages	Limitations
Traditonal conventional antibiotic therapy		Well-characterised PK/PD. Established regulatory paths.	Resistance emergence. High R&D costs. Poor intracellular penetration.
Novel anti-infectives	Bacteriophages	High specificity. Self-replication at infection site. Low toxicity.	Narrow host range. Phage-resistance emergence. Poor intracellular penetration. Low stability. Regulatory hurdles.
	Monoclonal antibodies	High specificity. Low variability. Low resistance selection. Predictable PK.	High production costs. Poor intracellular penetration. Poor clinical translation. Risk of immunogenicity.
	Antimicrobial peptides	Broad spectrum. Low resistance selection. Partial intracellular penetration.	Risk of cytotoxicity. Limited intracellular penetration. Prone to proteolytic degradation. Regulatory hurdles.
	Nanocarrier delivery systems	Enhanced intracellular delivery. Controlled release. Protection of labile drugs.	Risk of cytotoxicity. Risk of premature leakage or burst release. Unclear long-term biosafety. Endosomal entrapment. Regulatory hurdles.
Microbiota-based therapies		Limit pathogenic invasion. Promote immune homeostasis. Promote barrier integrity.	Biosafety concerns. Strong context-dependence. Lack of standardisation.
RNA-based strategies		High specificity. Modulation of genes and regulatory RNAs. Low resistance selection.	Instability and nuclease degradation. Delivery hurdles. Risk of off-target effects. Poor clinical translation. Regulatory hurdles.
Drug repurposing		Shorter development time. Lower costs. Known safety profiles. Unveiling new targets. Possibility of intracellular penetration.	Intellectual-property barriers. Limited commercial incentive. Risk of off-target effects. Slow clinical translation. Risk of unsafe dosing *in vivo*.

Abbreviations: PK, pharmacokinetics; PD, pharmacodynamics.

For intracellular infections, therapeutic efficacy depends on overcoming a series of barriers that are less relevant in purely extracellular disease. First, active compounds must reach infected host cells without unacceptable host toxicity, because intracellular pathogens exploit host-cell entry routes, immune signalling, cell-death pathways and metabolic rewiring that can also be perturbed by therapy (Kember et al., 2022; Uribe-Querol and Rosales, 2017; Watson et al., 2020; Peyrusson et al., 2020; Kamaruzzaman et al., 2017; Missiakas and Winstel, 2021; Bravo-Santano et al., 2018; Berkova et al., 2025; Terraza-Silvestre et al., 2025). Second, active compounds must accumulate in the correct subcellular niche, which may include acidic phagolysosomes, modified pathogen-containing vacuoles, damaged LC3-positive compartments or the cytosol (Flannagan et al., 2012; Westman and Grinstein, 2021; Buckley et al., 2016; Guan and Liu, 2020; Hernandez et al., 2004; Campoy and Colombo, 2009; Augenstreich et al., 2019; Nasiri and Venketaraman, 2025; Volk et al., 2024; Weinrick et al., 2004; Schwartz and Allen, 2006; Baxt and Goldberg, 2014; Wozniak et al., 2020; Ribet and Cossart, 2018; Hurley, 2015; Rivera-Cuevas and Carruthers, 2023; Jung et al., 2009; Franco et al., 2017; Nair et al., 2010; Missiakas and Winstel, 2021; Bravo-Santano et al., 2018). Third, they must retain activity against bacteria that often display slow growth, metabolic adaptation or persister-like phenotypes within host cells (Watson et al., 2020; Peyrusson et al., 2020; Bär et al., 2022; Huemer et al., 2021; Wong Fok Lung et al., 2019; Volk et al., 2024; Missiakas and Winstel, 2021; Bravo-Santano et al., 2018). Finally, candidate therapies should be validated in infection models that measure intracellular bacterial burden, subcellular localization, host-cell viability and, where possible, intracellular PK/PD, rather than relying exclusively on extracellular MICs (Peyrusson et al., 2020; Kamaruzzaman et al., 2017; Bravo-Santano et al., 2018; Deplanche et al., 2019; Deplanche et al., 2015; Alekseeva et al., 2013). We therefore discuss the therapeutic strategies below in relation to these barriers: host-cell entry, subcellular delivery, activity against intracellular or persistent bacteria, host toxicity and validation in intracellular infection models. These intracellular therapeutic barriers and the corresponding intervention and validation strategies are summarized in Table 2.

**TABLE 2:** Key therapeutic barriers in intracellular bacterial infections and corresponding intervention strategies.

Therapeutic barrier	Relevance to intracellular infection	Representative intervention strategies	Recommended validation endpoints
Host-cell penetration	Extracellular potency does not ensure adequate exposure within infected host cells	Cell-penetrating peptides; cyclotides; lipid or polymeric nanocarriers; optimized repurposed compounds	Cellular uptake and intracellular drug concentration; intracellular CFU or reporter-based efficacy assays
Subcellular delivery	Acidic phagolysosomal, vacuolar and cytosolic niches impose distinct delivery constraints	pH-responsive or lysosomotropic formulations; endosomal-escape systems; organelle-targeted carriers; modulators of autophagy or vesicular trafficking	Colocalization with LAMP1, LC3 or galectins; compartment-specific reporters; subcellular drug quantification
Activity against slow-growing or persistent bacteria	Intracellular adaptation can reduce bacterial growth and increase phenotypic antibiotic tolerance	Persister-active agents; metabolic sensitization; host-directed therapies; rational combination regimens	Intracellular time-kill assays; activity against SCVs or persisters; regrowth and relapse assays
Host-cell toxicity and therapeutic selectivity	Host-directed or membrane-active interventions may perturb essential host functions	Selective pathway modulation; targeted delivery; dose and exposure optimization; toxicity-sparing combinations	Host-cell viability and membrane integrity; mitochondrial function; inflammatory responses; therapeutic index
Model validity and translation	Conventional MIC testing does not capture host-cell penetration, subcellular distribution or intracellular PK/PD	Relevant infected-cell models; primary cells and organoids; professional and non-professional phagocytes; *in vivo* confirmation	Intracellular PK/PD; bacterial burden; host-cell damage; subcellular localization; persistence or relapse *in vivo*

Abbreviations: CFU, colony-forming units; LAMP1, lysosome-associated membrane glycoprotein 1; LC3, microtubule-associated protein 1 light chain 3; MIC, minimum inhibitory concentration; PK/PD, pharmacokinetics/pharmacodynamics; SCVs, small-colony variants.

### 4.1 Novel anti-infectives

Currently, the antibiotic pipeline is stagnating, with only 13 new drugs approved between 2017 and 2023 (World Health Organization, 2023). Natural compounds are a promising source for the discovery of new antimicrobials (Seukep et al., 2023). Some secondary metabolites, such as flavonoids, have been demonstrated to possess antimicrobial applicability (Liu Y. et al., 2025). However, compounds of natural origin often display poor bioavailability and face several challenges, including the loss of the source, difficulties in extraction and the presence of additional toxic components (Liu Y. et al., 2025; Ayon, 2023). Further antibiotic discovery also encompasses adjuvants that potentiate antibiotic activity and nanoformulations that improve delivery to intracellular targets (Melander and Melander, 2017; Soares et al., 2018; Mamun et al., 2021; Sodhi et al., 2023). Conventional antibiotic discovery has relied mainly on high-throughput and target-based screening, although the former produced most of the antibiotic classes used today (Inglese et al., 2007; Cox et al., 2017; Lewis, 2020). On the other hand, target-based screening can identify potent target inhibitors, but whole-cell activity is often limited by permeability and efflux (Payne et al., 2007; O’Shea and Moser, 2008). Hits from these screenings are sometimes prefiltered using Lipinski’s “rule of five” for solubility and permeability, then enter hit-to-lead development. In this phase, medicinal chemistry and structure–activity relationship studies generate analogues that are evaluated for toxicity and ADME (absorption, distribution, metabolism and excretion) properties; if they pass this stage, they can be considered leads, *i.e*., active, drug-like compounds selected for further optimisation toward a therapeutic candidate (Lipinski, 2000; Lipinski and Hopkins, 2004; Miethke et al., 2021). However, despite these efforts, hit identification remains low, with rates below 0.1%, and only about 5% of hits eventually become leads (Stanley et al., 2012; Roemer and Boone, 2013; Liao et al., 2018; Cardona et al., 2025).

Recent advances aim to improve efficiency through computational tools. Virtual screening, enabled by artificial intelligence and library data, can prioritize compounds with predicted antibacterial activity (Hochreiter et al., 2018; Rahman et al., 2022). Supervised machine-learning models use labelled datasets with binary or continuous outcomes linked to descriptors or SMILES (simplified molecular input line entry system) strings, while deep-learning architectures, such as directed message-passing neural networks, can learn features directly from chemical structures (Cardona et al., 2025; Rahman et al., 2022; Tarca et al., 2007). These approaches have contributed to the identification of halicin, abaucin, and PHAR A, and cultivation-independent strategies have also yielded compounds such as teixobactin (Rahman et al., 2022; Stokes et al., 2020; Theuretzbacher et al., 2023; Ling et al., 2015).

Despite these advances, antibiotic discovery remains challenging because of high costs, low profitability, long development timelines, complex regulatory approval, and limited clinical guidance (Clardy et al., 2006; McDevitt and Rosenberg, 2001). Moreover, in the race to discover new treatments, most new agents are still evaluated primarily under extracellular conditions, overlooking intracellular lifestyles. Despite all this, non-antibiotics are becoming a more attractive investment, since within the current clinical antibacterial pipeline, approximately 40% are non-traditional antibiotics (Gigante et al., 2022).

Among non-traditional antibiotics are bacteriophage therapies, monoclonal antibodies, and antimicrobial peptides. Phage therapies offer several advantages, since bacteriophages can be highly specific, are relatively easy to isolate, self-replicate at the site of infection, and, in general, have few reported side effects (Nikolich and Filippov, 2020; Śliwka et al., 2022). However, their specificity may complicate treatment of heterogeneous bacterial populations, and production and maintenance are expensive, with regulatory pathways being complex (Nikolich and Filippov, 2020; Śliwka et al., 2022). And, despite phage uptake having been reported (Bodner et al., 2021; Goswami et al., 2021), they present poor cell entry (Śliwka et al., 2022; Nieth et al., 2015). Their use against established intracellular reservoirs therefore remains constrained unless delivery systems or host-cell uptake strategies can reliably place active phage particles in the infected compartment.

On the other hand, monoclonal antibodies offer several advantages, including high specificity for essential or non-essential antigens, lower risk of selecting resistant mutants, low lot-to-lot variability, and high activity per unit mass of protein (Saylor et al., 2009; La Guidara et al., 2024). Antibacterial monoclonal antibodies can either enhance immune responses or neutralize virulence factors and toxins as their main mode of action (Nagy et al., 2017). Some monoclonal antibodies have shown intracellular activity, including the promotion of phagolysosome maturation, yet most candidates fail to control infection severity in preclinical and clinical trials (La Guidara et al., 2024; Vacca et al., 2022). Therefore, despite recent advances, the intracellular lifestyle remains a major weakness for these therapies, as antibodies rarely cross host cell membranes efficiently (La Guidara et al., 2024). Thus, their relevance for intracellular infections may be greatest when they prevent invasion, neutralize extracellular toxins or enhance phagolysosomal clearance, rather than as direct eradicators of established intracellular reservoirs.

Additionally, antimicrobial peptides are mainly pore-forming molecules that target and disrupt bacterial membranes, although additional intracellular targets have also been described (Ho et al., 2023; Wong et al., 2023). Some examples of antimicrobial peptides include lipopeptides, depsipeptides and the recently described odilorhabdins, which constitute a distinct class of ribosome-targeting antibiotics (Zeng et al., 2023; Saiyam et al., 2024; Racine and Gualtieri, 2019). They frequently possess a linear, amphipathic, and arginine-rich structure, usually cationic, but they often display limited resistance to proteolytic degradation (Mourenza et al., 2023; Sonawane et al., 2011; Chairatana and Nolan, 2014; Buccini et al., 2021). However, despite several antimicrobial peptides with intracellular antibacterial effects having been reported, only a minority are able to kill intracellular bacteria, and even fewer manage to efficiently internalise into host cells (Duarte-Mata and Salinas-Carmona, 2023). There have been several approaches to improve antimicrobial peptide activity. One attractive strategy is the use of D-enantiomeric peptides, in which natural L-amino acids are substituted with D-amino acids. D-enantiomers often show markedly improved proteolytic stability, enhanced antimicrobial potency and reduced toxicity compared with their L counterparts (Mendes et al., 2026; Sarkar et al., 2024; Jia et al., 2017; Zhao et al., 2016). In some cases, they also display better and more selective activity against Gram-positive bacteria (Ye and Aparicio, 2022; Zou et al., 2025). This approach has also shown promise against intracellular bacteria, although its use is still limited to a few preclinical examples, such as all-D peptides that inhibit intracellular *M. tuberculosis* in macrophages (Lan et al., 2014).

Within antimicrobial peptides, there is a multifunctional class known as cyclotides: small, naturally occurring cysteine-rich peptides, with a backbone cyclized head-to-tail and stabilized by a cysteine-knot motif, and which are arising as promising anti-infective scaffolds (Mourenza et al., 2023). Cyclotides offer several advantages. They can cross cell membranes and therefore act on both extracellular and intracellular bacteria (Mourenza et al., 2023; Contreras et al., 2011; Cascales et al., 2011). In addition, they are naturally resistant to proteolytic degradation, which confers markedly increased stability compared with many linear antimicrobial peptides (Rosengren et al., 2003). Antibacterial mechanisms of cyclotides have not yet been fully elucidated (Toke, 2005). However, the true strength of cyclotides lies in their engineerability. By grafting antimicrobial sequences onto the cyclotide framework or systematically modifying surface residues, researchers have generated stable cyclotides with potent antibacterial activity (Mourenza et al., 2023; Jacob et al., 2022). Therefore, cyclotides can be used as modular scaffolds: grafting other antimicrobial peptide sequences onto the cyclotide framework, combining the antimicrobial activity of the inserted peptide with the enhanced stability of the cyclotide backbone (Chaudhuri et al., 2019). In line with this, grafting membrane-active antimicrobial peptide sequences into the cyclotide scaffold yields engineered cyclotides that behave as membrane-targeting antimicrobials while gaining markedly improved proteolytic stability (Koehbach et al., 2021; Ganesan et al., 2021). Despite its high promise, this technology remains emergent and faces significant challenges, including cytotoxicity in some sequences, synthetic and production bottlenecks, and regulatory hurdles for clinical development (Cândido et al., 2025).

As can be seen, one of the main hurdles in treating intracellular bacterial infections is the limited penetration of host cell membranes. To overcome this barrier, drug delivery systems have been developed to enhance antimicrobial efficacy (Abed and Couvreur, 2014). These systems, often classified as organic, inorganic or hybrid nanoparticles, must cross host cell membranes, escape phagolysosomes and reach the bacterial niche to release their cargo, which makes them highly dependent on their physicochemical properties, such as size, charge, surface chemistry and shape (Chen Y. et al., 2023; Feng et al., 2023). Nanocarriers for intracellular infections must balance safety and biodegradability with high drug loading and controlled release to prevent premature leakage or burst effects (Hosseini et al., 2022). Metal nanoparticles, liposomes and cell-penetrating peptides illustrate the potential to enhance intracellular delivery, but each faces issues such as encapsulation instability, endosomal trapping, cytotoxicity and unresolved biosafety concerns, which still limit clinical translation (Chen Y. et al., 2023; Hosseini et al., 2022; Olowu et al., 2025).

### 4.2 Microbiota-based modulation of susceptibility to intracellular infection

The microbiota plays a central role in preventing a broad array of diseases, including infections (Ubeda et al., 2017). In the context of intracellular pathogens, its relevance is mainly linked to the early stages that precede or favour intracellular persistence, such as pathogen colonization, epithelial attachment, invasion, barrier disruption and local immune tone. Commensal bacteria counteract pathogens through multiple mechanisms; for example, *Staphylococcus lugdunensis*, a human commensal residing in the nares, produces lugdunin, an antimicrobial peptide that reduces skin and nasal colonization by *S. aureus* in rodents (Zipperer et al., 2016). In the gastrointestinal tract, hypoxia is a primary filter for microbial colonization, with less than 1% oxygen in healthy colonic epithelium (Zheng et al., 2015). The microbiota helps maintain a gut environment that limits pathogen growth, while dysbiosis can increase luminal oxygen and metabolites that favour infection; commensals also compete for nutrients and produce bacteriocins that suppress pathogens, although these mechanisms can sometimes be co-opted by bacteria such as *L. monocytogenes* (Gillis et al., 2018; Shin et al., 2015; Singh and Koo, 2024; Leshem et al., 2020). Microbiota-derived metabolism helps restrict colonization by supplying vitamins, modulating pH and inflammation, and generating metabolites such as bile acids and short-chain fatty acids (SCFAs) that can suppress pathogen growth and virulence (Duarte-Mata and Salinas-Carmona, 2023; Leshem et al., 2020; Zafar and Saier, 2021; Di Rienzi and Britton, 2020; Horswill et al., 2001). For example, *Salmonella* spp. infection is influenced by commensal-derived secondary bile acids and SCFAs, which can modulate virulence, inhibit growth and reduce host-cell internalization (Gillis et al., 2018; Leshem et al., 2020; Quereda et al., 2016; Anjana and Tiwari, 2022; Deng and Wang, 2024). These effects are context-dependent, since the same metabolites can also act as environmental cues that promote virulence under certain conditions (Ubeda et al., 2017; Quereda et al., 2016).

Microbiota-derived signals also shape mucosal immunity by promoting antimicrobial peptide production, regulating the Th17/Treg balance and supporting IgA class-switching, all of which help limit pathogen attachment and invasion (Platten et al., 2019; Dempsey and Corr, 2022; Wei et al., 2023; Collins et al., 2023; Shin et al., 2002; Jacobson et al., 2018; Lawhon et al., 2002; Gantois et al., 2006; Taha-Abdelaziz et al., 2019). SCFAs, for example, drive B-cell differentiation and IgA class-switching by inhibiting histone deacetylases and enhancing histone acetylation at key regulatory loci, reinforcing mucosal immunity and limiting epithelial attachment and invasion by pathogens (Vaishnava et al., 2008; Vaishnava et al., 2011). These insights have motivated microbiota-based interventions such as prebiotics, probiotics, postbiotics, synbiotics and faecal microbiota transplantation, but their use remains mainly supportive and context-dependent, with benefits that must be weighed against possible effects on cancer and other host processes (Loonen et al., 2014; Atarashi et al., 2011; Atarashi et al., 2013; Frosali et al., 2015; Bhaskaran et al., 2018; Cao et al., 2012; Yang et al., 2020; Kim et al., 2016; Moor et al., 2017; Hill et al., 2014). Thus, in the framework of intracellular bacterial infection, microbiota-based approaches should be viewed primarily as modulators of colonization resistance, invasion risk, epithelial barrier function and mucosal immunity, rather than as established strategies for directly eradicating intracellular bacterial reservoirs.

## 5 Platforms to identify and validate intracellular infection targets

### 5.1 Engineered microbial and mammalian systems to dissect host-pathogen pathways and therapeutic targets

To address therapeutic needs, engineered biological systems are emerging as powerful discovery tools. Synthetic biology has developed as an interdisciplinary field that combines engineering principles with biology to construct living systems with defined functions, providing platforms to uncover mechanisms and targets involved in both disease and treatment (Gibson et al., 2017). This has been particularly exploited in microbes, where engineered bacteria can enhance antimicrobial production or sense defined metabolites to trigger downstream antibacterial responses (Gibson et al., 2017). One example is a modified *Escherichia coli* strain that, upon detecting quorum-sensing signals from *Pseudomonas aeruginosa*, releases bacteriocins that facilitate pathogen clearance (Gibson et al., 2017; Swanson et al., 2020).

Genetic engineering also broadens the scope of research tools beyond these “therapeutic circuits”. For instance, fluorescent *Mycobacterium marinum* stress-reporter strains have been used to map bacterial stress responses to different antibiotic classes and to monitor these responses during infection, allowing extrapolation of mode-of-action signatures to *M. tuberculosis* infection (Salminen et al., 2021). Modified strains and knockouts have been particularly informative for dissecting how pathogens respond to stress at the molecular level. For instance, *S. aureus* SOS reporter strains combined with SOS response knockouts revealed that the major route to SOS induction by many antibiotics is RexAB-mediated processing of DSBs, and that disabling this module re-sensitizes bacteria to multiple drug classes (Yang L. et al., 2023). Similarly, systematic use of knockouts plus *gfp*-reporters has identified upstream DNA-repair factors as essential for robust SOS induction, highlighting them as promising targets to potentiate both DNA-damaging and non-DNA-targeting antibiotics (Tvede and Rask-Madsen, 1989). Moreover, knockout mutants have been used to link oxidative stress to the SOS response and to the emergence of small-colony variants with increased tolerance and persistence (Lawley et al., 2012). At a broader scale, CRISPR interference screens and other direct selection approaches have uncovered many genes not previously associated with antibiotic susceptibility (Deriu et al., 2013).

Engineered bacterial systems are also widely used for screening and classification of antimicrobial activities. For example, *B. subtilis* promoter reporter strains, in which transcription of specific stress response genes drives a fluorescent or luminescent readout, have been used to identify transcriptional inhibitors with activity against *S. aureus* (Bravo Santano et al., 2020). Similarly, genetically engineered *E. coli* reporter panels that differentially respond to inhibition of antibiotic-induced stress have been applied to detect and classify antibiotics according to their primary cellular target (Lorente-Torres et al., 2025). Library-based approaches leverage such strains for high-throughput screening, exemplified by cell-based infection screens in *S.* Typhimurium that identify host- or pathogen-directed hits (Huang et al., 2025). Reporter strains and knockouts in cell-based infection assays also help characterize how specific host functions are altered by pathogenic bacteria (Deplanche et al., 2019). Moreover, appropriately designed reporters can access difficult-to-treat phenotypes. For example, an *S. aureus* GFP reporter expressed only in metabolically active cells was used to identify KL1, a host-directed compound that re-sensitized persisters to antibiotic killing (Saeidi et al., 2011).

Although synthetic biology originally focused largely on bacteria, there is a growing shift towards engineered mammalian cells in infection biology (Gibson et al., 2017). Mammalian cell lines can be engineered to carry fluorescent reporters or stable knockdowns, enabling direct observation of host pathways in response to infection. mCherry–LC3 reporters in HeLa cells, for example, have been used to monitor autophagy dynamics upon infection with *E. coli* and *S. aureus* (Bravo-Santano et al., 2018; Boot et al., 2018). Other engineered lines allow tracking of intracellular bacterial localization to specific compartments (Bravo-Santano et al., 2018; Clarke et al., 2019; Ledger et al., 2023). Systematic knockdowns in host cells have revealed host genes and proteins exploited by intracellular pathogens, highlighting potential host-directed targets (Clarke et al., 2019). Additional mammalian reporters discriminate how host cells respond differentially to pathogenic *versus* non-pathogenic bacteria (Painter et al., 2015), or monitor host-cell viability in infection models (Hill et al., 2014; Ledger et al., 2023; Liu et al., 2024).

Despite the broad scope of these platforms, host-cell engineering remains comparatively underused, and integrated strategies combining engineered bacteria with engineered mammalian cells to modulate host pathways in the context of infection remain rare. Expanding such approaches could enhance efforts to unravel complex host-pathogen interactions and to identify therapeutic targets. Together with multi-omics approaches, these engineered systems provide complementary platforms to uncover host-directed targets and generate testable hypotheses for host-directed therapies.

### 5.2 Multi-omics and computational approaches to prioritise intracellular host-directed targets

For intracellular infections, multi-omics and computational approaches are most useful when they identify host or bacterial nodes that explain intracellular entry, subcellular niche adaptation, antibiotic tolerance, host-cell damage or persistence. Multi-omics can be defined as the combined application of high-throughput techniques that profile different molecular layers, including genomics, transcriptomics, proteomics and metabolomics, to capture biological dynamics that cannot be resolved by a single data layer alone (Lam et al., 2024). In host-pathogen interactions, this integration can expand the repertoire of druggable targets and repurposing opportunities, provided that candidate nodes are subsequently tested in intracellular infection models (Melamed et al., 2012). For example, proteomic and genomic analyses linked the natural product ecumicin to ClpC1 as a plausible target in *M. tuberculosis*, illustrating how omics can connect intracellular pathogen biology with druggable mechanisms (Adani et al., 2026). Important limitations remain, including cost, sampling variability, missing-value handling, heterogeneity between layers, interpretation, annotation, storage and computing requirements (Lam et al., 2024).

Multi-omics approaches are particularly well-suited to intracellular infections, which rewire multiple host pathways. At the metabolic level, macrophages shift from predominantly oxidative metabolism to aerobic glycolysis upon infection, with citrate and other TCA intermediates sustaining inflammatory responses under type I IFN and NF-κB control (Hsieh et al., 2020; Jha et al., 2015; Bhagwat et al., 2025). *Staphylococcus aureus*, for example, has been observed to extensively reroute host central carbon metabolism in non-phagocytic cells. These observations unveiled a starvation-dependent autophagy with increased nutrient uptake by the bacteria and depletion of intracellular reservoirs, thereby highlighting AMPK and ERK pathways as host targets to halt intracellular proliferation (Bravo-Santano et al., 2018). Using a phosphoproteomic approach, EPHA2, EGFR, ERBB2, c-Jun and N-WASP were identified as central host factors whose inhibition impairs entry and intracellular survival of *S. aureus* (Ledger et al., 2023). Some immunometabolites, such as itaconate and succinate, exert direct antibacterial pressure and stabilise HIF-1α, while infection-driven shifts in lipid profiles, including increased docosanoic, icosanoic and palmitic acid upon *S. aureus* infection, exert cytoprotective and regulatory roles (Bhagwat et al., 2025; Tannahill et al., 2013; Michelucci et al., 2013; Bravo-Santano et al., 2019a; Quehenberger et al., 2011). These examples illustrate how multi-omics can reveal host metabolic nodes which are manipulated or disrupted by intracellular bacteria and can serve as therapeutic targets.

Moreover, infection manipulates DDR and the epigenetic signature. Infection-associated DNA lesions activate DDR and checkpoint pathways that place the host cell at a crossroads between repair, senescence and death (Tong et al., 2024; Berkova et al., 2025; Tiwari and Wilson, 2019). This genotoxic stress is coupled to epigenetic reprogramming, with aberrant DNA methylation and histone post-translational modifications reshaping immune-gene expression and barrier integrity (Berkova et al., 2025; Millán-Zambrano et al., 2022; Wu et al., 2023). For example, Heppert et al. compared germ-free and conventional mice and revealed substantial changes in epithelial DNA methylation, underscoring the role of bacteria in shaping the epigenetic landscape (Heppert et al., 2021). In the context of *S. aureus*, transcriptome-wide profiling of infected osteoblasts uncovers strong inflammatory responses together with metabolic and epigenetic dysregulation, while airway epithelial cells exhibit altered DNA methylation and histone marks linked to chronic airway inflammation (Pérez-Novo and Bachert, 2015; Nicolas et al., 2022). Mechanistically, lactate-mediated HDAC11 inhibition and IL-10 induction provide a concrete example of how metabolic shifts, chromatin remodelling and cytokine expression are intertwined in a single multi-layered circuit (Berkova et al., 2025; Modak et al., 2014; Heim et al., 2020; Nicolas et al., 2022; Howden et al., 2023). These studies put DDR response as a host regulatory node that could be druggable for Host-directed therapies (HDTs) or repurposed drugs.

Nutritional immunity adds further layers that benefit from multi-omics integration. Host cells restrict access to iron, zinc, manganese and copper while rewiring TCA-derived metabolites and glycolysis as antimicrobial tools, thereby coupling metal homeostasis to immunometabolism (Bhagwat et al., 2025; Ezzeddine and Ghssein, 2023; Parrow et al., 2013). In response, intracellular pathogens deploy high-affinity uptake systems, such as siderophores, metallophores and dedicated transporters, and adopt flexible carbon-use strategies to survive within these constraints (Olive and Sassetti, 2016; Parrow et al., 2013; Choudhury et al., 2021; Mohsen et al., 2023). Multi-omics datasets that combine host and pathogen transcriptomes, proteomes and metabolomes can therefore reconstruct this landscape and highlight enzymatic steps, transporters or regulatory hubs amenable to pharmacological intervention. At the organelle level, many intracellular bacteria extensively remodel mitochondrial dynamics, respiration and quality control, perturbing fission-fusion balance and mitophagy (Mukherjee and Dikic, 2022; Escoll et al., 2021; Lu et al., 2025). Toxins such as VacA and LLO increase mitochondrial outer-membrane permeabilization, driving cytochrome c release, ROS production and mitochondrial DNA leakage that ultimately activate inflammasomes and TLR-linked inflammatory cascades (Mukherjee and Dikic, 2022; Galmiche and Rassow, 2010; Juliana et al., 2012). In *L. pneumophila* infection, the effector LpSpl mimics host S1P-lyase and degrades sphingosine-1-phosphate, altering sphingolipid pools and blocking starvation-induced autophagy, suggesting that boosting S1P signalling or inhibiting LpSpl activity could restore autophagic control of intracellular bacteria (Collins and Huett, 2018; Bravo-Santano et al., 2019b). Here, phosphoproteomics and quantitative proteomics, integrated with transcriptomics and metabolomics, offer a way to map these signalling pathways in detail and to prioritise kinases, phosphatases, epigenetic regulators or BCL-2 family members as targets for HDTs.

From a data-science perspective, multi-omics relies on bioinformatic pipelines for preprocessing (normalisation, batch correction and imputation of missing values) and on statistical and machine-learning frameworks to integrate heterogeneous datasets generated under different conditions and time points (e.g., similarity-network fusion, matrix factorisation, multi-block regression) (Lam et al., 2024; Bravo-Santano et al., 2019c). Network-based methods then map omics changes onto prior knowledge graphs of genes, proteins and metabolites to identify central hubs and modules associated with infection or drug response, while virtual screening, molecular docking and structure-based modelling link these nodes to existing or novel small molecules (Phillips et al., 2022; Castañera et al., 2026). Together, these bioinformatics approaches turn multi-layer measurements into ranked lists of candidate biomarkers, drug targets and repurposable compounds (Lam et al., 2024; Phillips et al., 2022).

When interpreted through an intracellular-infection framework, recent multi-omics studies across diverse pathogens illustrate four broad classes of druggable nodes: (i) metabolic hubs, (ii) DDR/epigenetic regulators, (iii) nutritional-immunity and metal-handling pathways, and (iv) organelle-linked signalling circuits. As a boundary example, microbial profiling combined with tumour transcriptome sequencing and metabolomics in glioma detected six enriched bacterial genera, including *Fusobacterium nucleatum*, as a potential diagnostic marker and putative therapeutic target; however, such tumour-associated signatures require functional validation before being interpreted as intracellular reservoirs or direct targets for intracellular infection therapy (Chen C. et al., 2023). Persistent *Brucella* spp. infection shifted host metabolism toward aerobic glycolysis and highlighted *de novo* nucleotide biosynthesis, especially inosine-5′-monophosphate dehydrogenase 2 (IMPDH2), as a key host hub (Nandi et al., 2020). Zimmermann et al. used dual RNA-seq and metabolomics in *M. tuberculosis*-infected macrophages to identify fewer genes truly essential for infection, narrowing down therapeutic targets and pointing to metabolic perturbations as druggable nodes (Chang and Kwon, 2016). In *L. monocytogenes*, transcriptomics and genome-scale metabolic modelling identified 12 metabolic pathways essential for intracellular replication, with deletions in these routes impairing intracellular growth; a core route involves branched-chain amino acid metabolism, whose limitation inhibits the CodY–PrfA virulence axis (Escoll et al., 2017). A genome-scale meta-analysis of host transcriptomic responses to *S. aureus* infection identified hubs in immune and stress pathways and prioritised bacteria-platelet interactions, apoptosis, autophagy, iron metabolism and ferroptosis for further host-directed investigation (Rolando et al., 2016). Using phosphopeptide enrichment and MS-based proteomics, Huemer et al. mapped the PknB-Stp axis as a druggable node against *S. aureus* persisters: acidic conditions resembling the intracellular milieu promote antibiotic tolerance and delayed growth, associated with PknB-dependent phosphoregulation and Stp-controlled dephosphorylation (Sahr et al., 2017). Metabolomics of *S. aureus* exposed to human serum showed decreased susceptibility to daptomycin alongside altered central metabolism, underscoring how growth conditions define adaptive metabolic responses to antibiotics (Yang M. et al., 2023). An integrative omics study across several pathogens, including *S. aureus*, identified the Isd iron-scavenging system as a serum-induced virulence module; in *S. aureus*, serum strongly upregulated the Isd haem acquisition system, and IsdI was proposed as a conserved serum-induced fitness determinant and promising anti-virulence target (Tomazou et al., 2021). Transcriptomics in *L. monocytogenes* infection identified SIRT2 as a host component required for intracellular survival: bacteria recruit SIRT2 to chromatin to promote H3K18 deacetylation, thereby reducing infection-induced DNA damage and preserving host cell viability, while pharmacological SIRT2 inhibition increased DNA damage and reduced intracellular burden (Noumi et al., 2025). For *C. burnetii*, Dot/Icm effectors uncovered by bioinformatics and functional screens target host vesicular trafficking, apoptosis, cytoskeleton and immune signalling, highlighting these host pathways as druggable nodes for host-directed therapies (Li T. et al., 2025). In *C. trachomatis*, transcriptomics showed differential expression of chlamydial factors such as CT147 and *cpoS*, involved in lysosomal fusion evasion and host trafficking manipulation, alongside a host response in which upstream IFN-I production is dampened while ISGs and chemokines (e.g., CCL2, CCL5, CXCL10, CXCL2, CSF2) are upregulated to maintain cell survival and metabolic activity, potentially allowing evasion of inflammatory cell death (Yu et al., 2023). *Shigella flexneri* remodels host lipid homeostasis and lipid-droplet formation for its own survival; lipidomics revealed that enriching host cells with C18:0 and C18:1 fatty acids reduced invasion and intracellular load, postulating modulation of lipid homeostasis and lipid droplets as a druggable node (Zimmermann et al., 2017). Finally, modulation of formate metabolism emerged as pharmacologically interesting, since formate can influence both virulence traits and host immune responses during *S. flexneri* infection (Lobel et al., 2012).

Together, these examples show how multi-omics and data-driven analysis can turn complex host-pathogen rewiring into plausible druggable host nodes and repurposing opportunities across diverse intracellular bacteria. However, omics-derived candidates should be advanced as therapeutic targets only when they satisfy intracellular validation criteria: reduced intracellular bacterial burden, preserved host-cell viability, activity in relevant host-cell types, evidence of correct subcellular exposure and compatibility with antibiotic or host-directed combinations. In Supplementary Table S1, we highlight selected host targets detected by multi-omics approaches that exemplify their utility for prioritizing intracellular infection mechanisms and therapeutic hypotheses.

## 6 Host-directed therapies

HDTs, although still in their infancy, are promising strategies to counteract intracellular bacterial pathogens (Russell et al., 2025). Host pathways provide attractive therapeutic targets to limit replication and persistence, as well as controlling dysregulated host responses and thus reducing inflammation and tissue damage and confining microbial niches (Huemer et al., 2023). Although the concept is not entirely new, HDTs offer alternative ways to confront pathogenic bacteria and are considered less prone to classical resistance, since microbes would have to rewire their dependence on conserved host functions or evade the activated host defence mechanisms (Huemer et al., 2023). Therefore, a deeper understanding of host-pathogen interactions is essential for unveiling novel therapeutic targets in both the pathogen and the host (Somerville et al., 2022).

To uncover host targets for antibacterial HDTs, researchers have combined multi-omics and systems-biology approaches with large-scale functional screens (RNAi/shRNA, microarrays, chemical libraries) that systematically perturb host genes and monitor infection phenotypes. These strategies have been used to map host–pathogen interactors and identify host genes required for bacterial entry, intracellular survival and infection-induced metabolic rewiring, revealing targets such as mitochondrial import components (TOMM40/TOMM22), endoplasmic-reticulum factors like TRAM2 (and the SERCA inhibitor thapsigargin), and AMPK-dependent starvation-induced autophagy in MRSA-infected cells (Clarke et al., 2019; Russell et al., 2025; Mu et al., 2023; Eldridge and Hamon, 2021; Yuan et al., 2025; Olagoke et al., 2025). Building on these discoveries, HDTs now include immunomodulatory agents (for example, monoclonal antibodies to TNF or interleukins, vitamin D_3_, HDAC inhibitors such as sulforaphane), cytokines and vitamins that enhance phagocyte antimicrobial functions (cathelicidin, ROS, autophagy), and strategies that modulate hypoxia pathways (HIF-1α) or specialised lipid mediators (resolvins) to boost bacterial clearance while limiting tissue damage (Huemer et al., 2023; Ascari et al., 2023; Wang et al., 2023; Bravo-Santano et al., 2019d; Kaufmann et al., 2018; Schwegmann and Brombacher, 2008). Despite their promise, HDTs face important challenges: species differences in immune networks complicate translation, pleiotropic host targets raise toxicity concerns, and efficacy is highly stage-dependent, making biomarker-guided timing, patient stratification and integration into personalised regimens essential but also more complex and costly for healthcare systems (Huemer et al., 2023; Olagoke et al., 2025; Cheng et al., 2005).

### 6.1 miRNA and RNA-based therapies

Use of RNA-based therapies is becoming increasingly popular following the success of RNA vaccines (Derré et al., 2007). There are rapidly evolving approaches, including messenger RNA (mRNA), RNA interference (RNAi), antisense oligonucleotides, and small RNAs (<200 nucleotides in length) such as small interfering RNA (siRNA), piwi-interacting RNA (piRNA), and microRNA (miRNA) modulate gene expression, protein production, or cellular processes to address infectious diseases (Derré et al., 2007; Schweppe et al., 2015). RNA therapies refer to RNA molecules that can be used directly as therapeutics, while RNA-based therapies include RNA-dependent technologies like CRISPR-Cas (Chiang et al., 2018). In general, they can be sorted as antisense technologies, mRNA-based strategies, RNAi-based therapeutics (which include miRNAs), and CRISPR-Cas genome editing (Schweppe et al., 2015).

The use of short, single-stranded synthetic oligonucleotides that bind target RNAs for degradation or for blocking translation is the basis of antisense technologies, which enable precise modulation of host or pathogen function during infection (Wallis et al., 2009). In contrast, mRNA-based strategies encode therapeutic proteins (e.g., antigens, cytokines, antimicrobial enzymes), applicable as vaccines for intracellular bacteria (Schweppe et al., 2015; Wroblewski et al., 2010). Alternatively, CRISPR-Cas systems provide RNA-guided platforms to edit or silence host or bacterial genes involved in intracellular infections (Schweppe et al., 2015). Among RNAi-based therapeutics, synthetic siRNAs, shRNAs, and miRNA mimics that are loaded into RNAi machinery to silence genes, mimicking endogenous pathways that degrade RNA targets in response to viral infection or transposon activity (Liu et al., 2007). These versatile strategies possess lower immunogenicity than viral vectors, serving as accurate tools for targeting genes and/or proteins (Schweppe et al., 2015). Nonetheless, they still face challenges, such as susceptibility to nuclease degradation, rapid clearance, poor tissue penetration, and TLR-mediated immunogenicity (Schweppe et al., 2015). Delivery is therefore a major limiting factor, since unmodified oligonucleotides display limited stability, cellular uptake and endosomal escape (Yedery and Jerse, 2015). However, advances from antiviral RNA therapeutics, including chemical modification and nanoparticle-based carriers, have substantially improved intracellular delivery while reducing innate immune activation, and provide a framework that can be leveraged for antibacterial applications (Schweppe et al., 2015; Dalli et al., 2015; Seok et al., 2013; Mourenza et al., 2022; Singh et al., 2026; Liu M. et al., 2025; Crooke et al., 2021; Chen et al., 2025).

Until a flagellin-induced miR-393a in *Arabidopsis thaliana* upon *Pseudomonas syringae* infection was observed and a study in monocytes recorded miRNome changes in human monocytes, miRNAs were not linked to infection (Sparmann and Vogel, 2023; Maziec et al., 2025). Although originally identified as regulators of developmental processes, miRNAs also exert antimicrobial effects by silencing host genes that are essential for infection progression (Derré et al., 2007). Their tissue-specific expression is shaped by polymorphisms, DNA methylation, strand selection and interactions with RNAs and proteins (Heggie et al., 2025). Biogenesis begins with RNA polymerase II transcription of primary miRNAs, which are processed by DROSHA into pre-miRNAs that are exported to the cytoplasm by exportin-5; there, DICER generates mature miRNA duplexes that are loaded into the RNA-induced silencing complex (RISC) to target 3′-UTRs, typically repressing gene expression but also indirectly upregulating genes by degrading upstream repressors (Ingle and Fang, 2023; Paunovska et al., 2022; Friedrich and Aigner, 2022; Feng et al., 2021; Sung and Kim, 2020; Chow et al., 2020; Navarro et al., 2006).

During infection, miRNAs regulate the cell cycle, cytoskeleton, autophagy, cell death/survival and innate immunity, as illustrated by lipopolysaccharide stimulation of TLR4, which upregulates miR-155, miR-146a/b and miR-132 to establish a negative feedback loop that limits excessive TLR4 signalling (Maziec et al., 2025; Taganov et al., 2006). miR-155 is induced in several intracellular infections, including those caused by *Francisella tularensis*, *M. tuberculosis*, *Vibrio cholerae*, *S. aureus*, *L. monocytogenes* and *H. pylori*, and depending on the context it can either promote bacterial clearance or exacerbate pathology through inflammation, cytokine storms or autophagy, acting as a pro- or anti-inflammatory regulator that also modulates apoptosis and autophagy (Derré et al., 2007; Taganov et al., 2006; Correia de Sousa et al., 2019; Bernstein et al., 2001; Hutvágner et al., 2001; Lee et al., 2003; Bartel, 2004; Han et al., 2009; Yamazawa et al., 2018). Bacterial stimulation induces dynamic changes in host miRNA expression through innate immune signalling pathways (Taganov et al., 2006). Intracellular pathogens frequently converge on miR-155, miR-146a/b, let-7, miR-29 and miR-21 to dampen interferon and NF-κB signalling and thereby evade apoptosis (Derré et al., 2007; Taganov et al., 2006; Acuña et al., 2020). For example, *L. monocytogenes* upregulates miR-155, miR-146a and miR-125a while downregulating miR-145 and the let-7 family to attenuate type I IFN and inflammation; *Salmonella* spp. induces NF-κB-driven miR-155, miR-146 and miR-21 while suppressing let-7, a regulator of IL-6 and IL-10; *S. aureus* enterotoxin B causes miR-155 overexpression and IFN-γ excess; and *Mycobacterium leprae* uses miR-21 to escape antimicrobial peptides while recurrently downregulating let-7 (Taganov et al., 2006; Aguilar et al., 2019; Cremer et al., 2009; Bandyopadhyay et al., 2014; Podsiad et al., 2016; Rothchild et al., 2016; Bitar et al., 2019; Săsăran et al., 2021). More recently, high-throughput screening of miRNA mimics identified miR-4430, miR-1249-5p and miR-147a as miRNAs that reduce intracellular *S. aureus* burden and protect host cells, with transcriptomic analyses showing that these miRNAs enhance innate immune pathways and limit bacterial invasion (Liu et al., 2024). Therefore, miRNAs are emerging as non-invasive biomarkers for clinical diagnosis, and their therapeutic applications are only beginning to be explored (Wang et al., 2013; Maudet et al., 2014).

Since miRNA-based strategies have shown promise against viral infections (Chen et al., 2007; Iliopoulos et al., 2009), anti-miRNAs that target host miRNAs facilitating bacterial infection could, in principle, delay or even prevent pathogen colonisation (Derré et al., 2007). Anti-miRNAs are synthetic single-stranded RNAs complementary to specific miRNAs, designed to bind and inhibit their function (Schulte et al., 2011). Their major limitation is off-target activity, because they may also bind partially complementary mRNAs, leading to unintended effects (Izar et al., 2012; Liu et al., 2012). As with other RNA-based approaches, delivery poses an additional hurdle: anti-miRNAs are susceptible to nuclease degradation, prompting the development of diverse delivery systems in which exosomes have shown favourable safety profiles and, unlike adenoviral vectors, reduced susceptibility to neutralisation by host antibodies (Derré et al., 2007; Liu et al., 2012; Teng et al., 2013; Kumar et al., 2015). In addition, robust phenotypic effects generally require high endogenous expression of the targeted miRNA, so inhibition of weakly expressed miRNAs may have minimal impact on gene expression (Liu et al., 2024).

### 6.2 Drug repurposing

Drug repurposing is the process of identifying new therapeutic indications for existing drugs, most often FDA-approved compounds with well-characterised safety, toxicity, pharmacology and formulation profiles (Lawrie et al., 2008; Mitchell et al., 2008). Historically, it has occurred largely serendipitously in the absence of systematic discovery pipelines, as exemplified by sildenafil, which was originally developed as an antihypertensive before its unexpected effects led to its repositioning for erectile dysfunction and the opening of a major new market (Drury et al., 2017; Jamalkhah et al., 2021). The COVID-19 pandemic markedly accelerated interest in repurposing as a rapid route to treatment development, highlighting its value for emerging infectious diseases (Iannaccone et al., 2014; Wang et al., 2011). In this context, leveraging the extensive clinical and pharmacological data available for approved drugs makes repurposing an especially attractive strategy against intracellular bacteria (Deng et al., 2014; Pecot et al., 2011).

*De novo* drug development typically costs between 314 million and 2.8 billion USD and requires 10–17 years, whereas repurposing can shorten timelines to 3–12 years at roughly half the cost, with overall approval success rates exceeding 30% compared with about 10% for new molecular entities (Mathiyalagan and Sahoo, 2017; Konreddy et al., 2019; Quezada et al., 2020; Pushpakom et al., 2019). Over the past four decades, approvals of repurposed drugs have steadily risen, yet infectious diseases account for only 5.5% of these cases, compared with 13% of *de novo* approvals (Juárez-López and Schcolnik-Cabrera, 2021). Strikingly, only around 1% of the drug-repurposing literature focuses on intracellular pathogenic bacteria, underscoring both the neglect of this niche and the untapped potential of repurposing in this area (Pecot et al., 2011).

Recent advances in high-throughput screening, bioinformatics and systems biology have greatly facilitated the identification of repurposing candidates (Clarke et al., 2019; Ledger et al., 2023; Galindez et al., 2021). Drug repurposing strategies can be broadly divided into computational and experimental approaches, which tend to yield the best results when used in combination (Kulkarni et al., 2023). Machine-learning models that integrate molecular fingerprints, docking scores and multi-omics signatures have been used to prioritise compounds, with algorithms such as Random Forests, support vector machines and multilayer perceptrons successfully flagging novel antibacterial agents acting on previously underexplored pathways (Stokes et al., 2020; Ahmad et al., 2024). Structure-based virtual screening remains a central *in silico* method for identifying high-affinity ligands for defined targets, as illustrated by the repurposing of fenoprofen as an inhibitor of the *S. aureus* response regulator SaeR and by docking/molecular-dynamics studies showing lumacaftor binding to the *S. aureus* FemX enzyme involved in cell-wall synthesis (Rahman et al., 2022; Lorente-Torres et al., 2024). Signature-based matching represents another computational avenue, comparing drug-induced transcriptional or phenotypic profiles with disease signatures across multi-omics datasets, adverse-event reports and structural information; this strategy identified clofilium tosylate and glibenclamide (glyburide) as candidates capable of reversing *S. aureus* endophthalmitis gene-expression patterns (Jampilek, 2022; Wouters et al., 2020; Tiberi et al., 2018).

**FIGURE 2:**
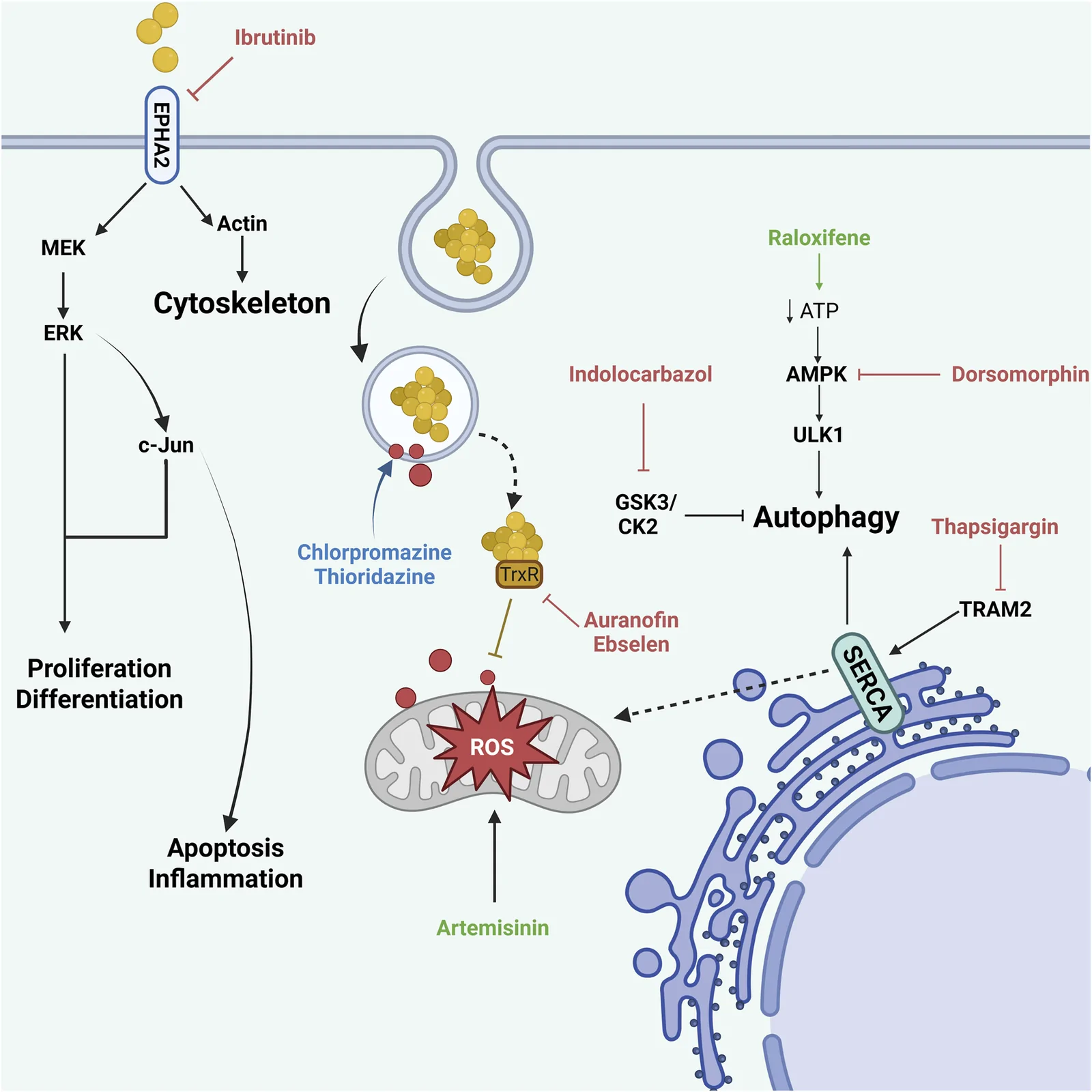
Host-directed therapeutic targets modulating intracellular infection. Schematic representation of host signalling pathways and cellular processes exploited during intracellular bacterial infection and targeted by repurposed drugs. EPHA2-ERK-cJun signalling regulates actin cytoskeletal remodelling, proliferation, differentiation, apoptosis, and inflammation; this axis can be pharmacologically targeted by Ibrutinib (EPHA2), MEK/ERK inhibitors, and other agents that affect actin dynamics. *S. aureus* encodes thioredoxin reductase (TrxR), which is essential for redox homeostasis and can help the bacterium to cope with host-derived ROS. Auranofin and Ebselen both target TrxR, compromising bacterial redox homeostasis and sensitizing against host-derived ROS, promoting intracellular clearance. Phenothiazines such as chlorpromazine and thioridazine accumulate in macrophages, increasing lysosomal activity and ROS production, and thereby enhancing the killing of intracellular *S. aureus* within phagolysosomal compartments. Artemisinin exerts antibacterial activity *via* ROS generation. Autophagy, another major host pathway hijacked by *S. aureus*, is controlled by AMPK-ULK1 signalling and calcium homeostasis. Some repurposed drugs modulate autophagy. Thapsigargin, dorsomorphin and indolocarbazoles inhibit autophagy by blocking the SERCA pump, inhibiting AMPK activity and targeting host kinases such as GSK3 and CK2, respectively, whereas raloxifene promotes autophagy and neutrophil survival. Collectively, targeting these pathways may determine bacterial clearance and host cell fate.

Despite their power to generate hypotheses, computational methods ultimately require experimental and clinical validation. Target-based screens, which interrogate either bacterial or host targets in a mechanism-driven manner, are widely used but limited by challenges in target selection, high false-positive rates and narrow biological scope (Novack and Moshiri, 2021; Akodad et al., 2026). Phenotype-based screens, in contrast, assess compound effects directly on cells or organisms without prior target knowledge, thereby capturing complex biology at the expense of demanding extensive follow-up to deconvolute off-target effects (Mourenza et al., 2020; Li Y. et al., 2025). *In vivo* models remain essential for preclinical validation but are constrained by high cost, ethical considerations, interspecies differences and reproducibility issues (Kulkarni et al., 2023). To date, repurposing efforts have yielded multiple candidates with potential utility against intracellular bacteria, ranging from older antimicrobials redirected toward new bacterial pathways to autophagy inducers and anticancer drugs with established intracellular activity (Figure 2) (Clarke et al., 2019; Ledger et al., 2023; Li et al., 2022).

Nevertheless, drug repurposing faces substantial obstacles. Intellectual-property constraints remain a primary barrier, since commercial viability often depends on securing new patents supported by positive clinical-trial data (Drury et al., 2017). Heavy reliance on public and academic funding further dampens private investment, particularly for off-patent compounds (Juárez-López and Schcolnik-Cabrera, 2021). Clinical translation is progressing slowly, with only a handful of trials (two ongoing and one completed) indicating that prior approval does not automatically accelerate authorisation for new indications (Iannaccone et al., 2014). Additional hurdles include trial failures, incomplete understanding of bactericidal mechanisms, uncertain resistance potential, limited funding and a diminished sense of urgency to develop therapies specifically targeting intracellular bacteria while conventional antibiotics remain effective for most patients (Jamalkhah et al., 2021; Pecot et al., 2011). Even so, repurposed agents offer attractive mechanisms of action, including immunomodulation, broad-spectrum or cross-species efficacy and control of multidrug-resistant pathogens *via* host-directed pathways, with host-targeted approaches in particular expected to impose lower selective pressure for resistance (Ledger et al., 2023; Galindez et al., 2021; Jiang et al., 2023; Keiser et al., 2009). The field is advancing rapidly but still needs to address issues such as bacterial resistance and tolerance, safety in patients with comorbidities and the optimisation of dosing regimens and formulations (Pecot et al., 2011; Mathiyalagan and Sahoo, 2017).

### 6.3 Combinatorial therapies

Host-directed therapies offer clear advantages but are unlikely to function optimally as stand-alone treatments, since their use in isolation can raise issues analogous to those seen with conventional antibiotic therapy (Olagoke et al., 2025). To more effectively confront multidrug resistance, combination regimens—at their simplest, the use of two or more drugs in tandem—have emerged as particularly promising (Olagoke et al., 2025; Rajamani et al., 2016). In this setting, repurposed agents combined with standard antibiotics can extend the useful lifespan of existing drugs, reduce the likelihood of therapeutic escape by bacteria sheltering inside host cells and shorten treatment courses in chronic or relapsing infections (Russell et al., 2025). Classic combinations have mainly paired two antibiotics, an antibiotic plus a resistance-targeting compound or an antibiotic with a potentiating adjuvant, but dual-HDT regimens may be especially advantageous when at least one component also exerts direct bactericidal activity (Olagoke et al., 2025; Das et al., 2022).

A well-known example is the trimethoprim–sulfamethoxazole combination, which sequentially inhibits folate biosynthesis, allowing dose reduction, delaying resistance and improving clinical outcomes relative to either drug alone (Matilla et al., 2018; Tucker et al., 2018). Many of the most successful regimens exploit pharmacodynamic synergy, where combined agents achieve an effect greater than the sum of their individual activities. In *S. aureus*, for instance, clomiphene, selamectin and crizotinib (originally developed as a fertility drug, an anthelmintic and an anticancer agent, respectively) have shown synergistic activity with standard antibiotics (Ledger et al., 2023; Cheng et al., 2019; Le et al., 2020; Chifiriuc et al., 2022). In *M. tuberculosis*, simvastatin enhances isoniazid and rifampicin; metformin acts synergistically with statins; carbapenems combined with rifampin, doripenem, or biapenem improve efficacy, and chlorpromazine together with gatifloxacin also displays synergy (Pecot et al., 2011; Zumla et al., 2015; Shapira et al., 2024; Haldar, 2025; Kilinç et al., 2021).

Using clinically approved drugs in combination with conventional antibiotics thus brings several advantages, including extending antibiotic lifespan and shortening treatment duration, particularly in chronic or intracellular infections (Ledger et al., 2023; Olagoke et al., 2025). For such regimens to be effective *in vivo*, however, repurposed components often need to achieve unbound plasma trough concentrations at clinically acceptable doses that exceed their *in vitro* EC50 or MIC in combination settings (Russell et al., 2025). Many repurposed agents primarily act as adjuvants that restore or enhance antibiotic activity, thereby lowering the selective pressure associated with high-dose monotherapy and reducing the risk of resistance evolution (Masters et al., 2003). More broadly, combination therapy has become a viable strategy across multiple infectious diseases, as carefully selected drug pairs and dosing schedules can decrease overall drug burden while maintaining efficient clearance of intracellular reservoirs (Kulkarni et al., 2023; Dhanda et al., 2023). These regimens can extend antibiotic utility, shorten therapy, improve bacterial eradication and exploit synergistic interactions, particularly when HDTs target complementary signalling pathways (Das et al., 2022; Dhanda et al., 2023). Nonetheless, they are not without drawbacks: achieving effective unbound concentrations for all components, managing increased toxicity and more complex pharmacokinetics/pharmacodynamics, and uncertainty about resistance selection in pathogens and, to a lesser extent, host adaptation, remain important challenges (Russell et al., 2025; Das et al., 2022).

## 7 Limitations and caveats of current strategies

Given the current approaches discussed in this review, several limitations must be considered when translating conceptual advances into effective treatments for intracellular infections. Most of these strategies are still in their infancy and require further development. Engineered living therapeutics continue to face biosafety concerns and regulatory hurdles that are only beginning to be addressed, and experimental platforms remain simplified, relying on controlled conditions that do not fully capture the complexity of host-cell microenvironments. Likewise, *in vivo* models are constrained by interspecies differences, cost and reproducibility issues, which limit direct extrapolation to human disease. Multi-omics and *in silico* approaches, despite providing a broad system-level view of host-pathogen interactions, remain expensive, heterogeneous and largely hypothesis-generating, and therefore require extensive functional validation. Host-directed targets, although attractive and promising, may also produce undesired effects if not carefully selected and timed, and bacteria may still adapt to such interventions. Combination regimens further increase complexity by adding pharmacokinetic and toxicity challenges and by requiring careful optimisation of dosing and scheduling in already vulnerable patients.

These scientific challenges converge with clinical translation issues shaped by economic and regulatory hurdles. Antibiotic discovery suffers from low hit-to-lead success rates, high development costs and limited commercial incentives, whereas non-traditional approaches face complex regulatory pathways and standardisation issues. At present, most non-traditional antibacterial approaches are being developed predominantly in academic settings and early-stage public–private partnerships, with comparatively limited late-stage industrial investment owing to regulatory complexity, high perceived risk and uncertain return on investment. Together, these caveats underscore that many promising strategies against intracellular bacteria are still in their early stages, and that progress will depend on integrating these approaches with robust mechanistic insight and multi-omics data, developing more predictive models that better reflect the complexity of these infections, and designing clinical and regulatory frameworks able to accommodate non-traditional anti-infective approaches.

## 8 Conclusions and future perspectives

Today, intracellular pathogenic bacteria remain a significant blind spot in clinical practice, even as the parallel rise of multidrug resistance poses an increasingly serious threat. There is an urgent need to reframe bacterial infection by recognising the intracellular niche as an essential component of pathogenesis, since host-pathogen interactions ultimately determine infection outcome. Host cellular processes have emerged as attractive therapeutic targets, and a new therapeutic landscape is taking shape, extending beyond *de novo* antibiotic discovery to include host-directed interventions, microbiota-based strategies, drug repurposing, non-traditional anti-infectives, and engineered biological platforms. Treating infections with rational combinations of these strategies may improve bacterial clearance and clinical outcomes, limit the emergence of resistance and shorten treatment duration. However, because many of these approaches are still emerging and supported mainly by preclinical data, they face scientific, regulatory and economic challenges that currently constrain their clinical translation.
