# Supplementary material for: Fighting the invader: strategies against intracellular bacteria
Source: Front Pharmacol. 2026 Aug 20;17:1904697. doi: 10.3389/fphar.2026.1904697 (PMC13539124; doi:10.3389/fphar.2026.1904697)
Supplement: Supplementary file 1 [file Table1.docx]

Supplementary Material

**Table S1. Host cellular targets and pathways identified by multiomics analyses across different intracellular bacterial pathogens.**

| Target | Pathogen | Pathway | Reference |
| --- | --- | --- | --- |
| Hexokinase | *M. tuberculosis* | Inhibition of IL-1beta Inhibition of glycolisis | (Odçikin *et al.*, 2002; Tannahill *et al.*, 2013; Zhong *et al.*, 2019; Kim, Kim and Yang, 2020) |
| Glucose transporters | *M. tuberculosis* | Inhibition of glycolisis | (Hresko and Hruz, 2011; Kim, Kim and Yang, 2020) |
| Pyruvate dehydrogenase kinase | *M. tuberculosis* | Inhibition of glycolisis | (Tataranni *et al.*, 2019; Kim, Kim and Yang, 2020) |
| Lactate dehydrogenase | *M. tuberculosis* | Inhibition of glycolisis Downregulation of cytokines and iNOS | (Song *et al.*, 2018; Kim, Kim and Yang, 2020) |
| Pyruvate kinase M2 | *M. tuberculosis* | Inhibition of HIF-1alpha Suppression of IL-1beta | (Palsson-McDermott *et al.*, 2015) |
| mTOR | *M. tuberculosis S. aureus* | Inhibition of glycolisis Upregulation of antimicrobial effect Modulation of autophagy | (Gutierrez *et al.*, 2004; Andersson *et al.*, 2016; Juárez *et al.*, 2016; Kim, Kim and Yang, 2020; Russell *et al.*, 2025) |
| AMP kinase | *M. tuberculosis* | Increased fatty acid oxidation Inhibition of antibacterial activity Reduced gene of inflammation | (Singhal *et al.*, 2014; Yang *et al.*, 2014; Rena, Hardie and Pearson, 2017; Kim, Kim and Yang, 2020) |
| Fatty acid synthase | *M. tuberculosis* | Inhibition of fatty acid synthesis Reduced the inflammation and oxidative stress Swithc M2 to M1 Downregulation of NLRP3 inflammasome | (Moon *et al.*, 2015; Idrovo *et al.*, 2016; Bose *et al.*, 2019; Kim, Kim and Yang, 2020) |
| PPARγ | *M. tuberculosis* | Modulation of lipid metabolism, inflammation and pathogenesis of bacteria | (Nicolaou, Goodall and Erridge, 2012; Kim, Kim and Yang, 2020) |
| PGC-1α | *M. tuberculosis* | Inhibition of NF-kB signalling and pro-inflammatory response. Upregulation of fatty acid oxidation and anti-inflammation | (Kauppinen *et al.*, 2013; Vachharajani *et al.*, 2016; Cheng *et al.*, 2017; Kim, Kim and Yang, 2020) |
| Serine dehydratase | *M. tuberculosis* | Valine, Isoleucine, Leucine biosynthesis | (Jiang *et al.*, 2021) |
| Serine dehydratase like | *M. tuberculosis* | Valine, Isoleucine, Leucine biosynthesis | (Jiang *et al.*, 2021) |
| BCAA transaminase 1 | *M. tuberculosis* | Valine, Isoleucine, Leucine biosynthesis | (Jiang *et al.*, 2021) |
| BCAA transaminase 2 | *M. tuberculosis* | Valine, Isoleucine, Leucine biosynthesis | (Jiang *et al.*, 2021) |
| Glycine-N-acetyltransferase | *M. tuberculosis* | Phenylalanine metabolism | (Jiang *et al.*, 2021) |
| Aldehyde-dehydrogenase 3 family member B1 | *M. tuberculosis* | Phenylalanine metabolism | (Jiang *et al.*, 2021) |
| IL4 induced 1 | *M. tuberculosis* | Phenylalanine metabolism Phenylalanine, tyrosine, tryptophan biosynthesis | (Jiang *et al.*, 2021) |
| Glutamic-oxoalacetic transaminase 1 | *M. tuberculosis* | Phenylalanine metabolism | (Jiang *et al.*, 2021) |
| Macrophage migration inhibitory factor | *M. tuberculosis* | Phenylalanine metabolism | (Jiang *et al.*, 2021) |
| VWF | *S. aureus* | Inhibition of fibrin/platelet microthrombus formation | (Russell *et al.*, 2025) |
| GP11b (ITGA2B) | *S. aureus* | Inhibition of fibrin/platelet microthrombus formation | (Russell *et al.*, 2025) |
| IL17RA | *S. aureus* | Bactericidal activity | (Russell *et al.*, 2025) |
| CD47 | *S. aureus* | Inhibition of phagocytosis and modulation of cell clearance | (Russell *et al.*, 2025) |
| ASGR2 | *S. aureus* | Inhibition of S aureus-induced platelet loss | (Russell *et al.*, 2025) |
| CD40 | *S. aureus* | Modulation of immune response | (Russell *et al.*, 2025) |
| NPC2 | *S. aureus* | Endolysosomal cholesterol transport Lipid trafficking  Modulation membrane composition | (Russell *et al.*, 2025) |
| BID | *S. aureus* | Modulation of apoptosis | (Russell *et al.*, 2025) |
| Prothrombin F2 | *S. aureus* | Coagulation cascade  Thrombin generation | (Russell *et al.*, 2025) |
| PDL1 (CD274) | *S. aureus* | Modulation of immune checkpoints | (Russell *et al.*, 2025) |
| B7-1 (CD80) | *S. aureus* | Modulation of immune response | (Russell *et al.*, 2025) |
| IFNγ | *S. aureus* | Bactericidal activity | (Russell *et al.*, 2025) |
| IL7R | *S. aureus* | Bactericidal activity | (Russell *et al.*, 2025) |
| PTAFR | *S. aureus* | Modulation of inflammation Platelet‑activating factor receptor Leukocyte recruitment | (Castañera *et al.*, 2026) |
| STAT1 | *S. aureus* | Modulation of interferon signalling | (Castañera *et al.*, 2026) |
| PARP12 | *S. aureus* | Modulation of immune response | (Castañera *et al.*, 2026) |
| INSIG1 | *S. aureus* | Modulation of sterol and lipid metabolism | (Castañera *et al.*, 2026) |
| ITGA5 | *S. aureus* | Fibronectin receptor integrin α5 Cell adhesion Bacterial invasion | (Castañera *et al.*, 2026) |
| FN1 | *S. aureus* | Fibronectin protein Extracellular matrix remodelling Cell adhesion  Bacterial binding. | (Castañera *et al.*, 2026) |
| HDAC11 | *S. aureus* | Histone deacetylase Epigenetic repression  IL‑10 regulation. | (Modak *et al.*, 2014; Heim *et al.*, 2020; Nicolas *et al.*, 2022; Howden *et al.*, 2023) |
| DNMTs | *S. aureus* | DNA methylation and long‑term gene silencing | (Pérez-Novo and Bachert, 2015; Nicolas *et al.*, 2022; Howden *et al.*, 2023; Berkova *et al.*, 2025) |
| AMPK | *S. aureus* | Energy sensor kinase Metabolic stress response  Modulation of autophagy | (Bravo-Santano *et al.*, 2018) |
| ERK | *S. aureus* | Growth factor signalling, proliferation and stress responses | (Bravo-Santano *et al.*, 2018) |
| EPHA2 | *S. aureus* | Modulation of cell cycle Pathogen entry | (Bravo-Santano *et al.*, 2019) |
| C-JUN | *S. aureus* | Stress response Modulation of inflammatory gene expression | (Bravo-Santano *et al.*, 2019) |
| ERBB2 | *S. aureus* | ERBB signalling Proliferation and survival | (Bravo-Santano *et al.*, 2019) |
| WASL (N‑WASP) | *S. aureus* | Actin cytoskeleton remodelling | (Bravo-Santano *et al.*, 2019) |
| EGFR | *S. aureus* | ERBB/MAPK signalling  Cytoskeleton remodelling | (Bravo-Santano *et al.*, 2019) |
| IFN-I/IRF9 | *L. monocytogenes* | Inflammatory and metabolic pathways of IFN-I | (Demiroz *et al.*, 2021) |
| SIRT2 | *L. monocytogenes* | Modulation of DDR | (Eldridge and Hamon, 2021) |
| IMPDH2 | *Brucella spp.* | Nucleotide biosynthesis | (Yu *et al.*, 2023) |
| DDX58 | *C. trachomatis* | RIG‑I RNA sensor Modulation of type I IFN response | (Olagoke, Chittaranjan and Dean, 2025) |
| IFIH1 | *C. trachomatis* | MDA5 RNA sensor Modulation of type I IFN response | (Olagoke, Chittaranjan and Dean, 2025) |
| IRF7 | *C. trachomatis* | Modulation of type I IFN response Modulation of ISGs | (Olagoke, Chittaranjan and Dean, 2025) |
| CCL5 | *C. trachomatis* | Leukocyte recruitment | (Olagoke, Chittaranjan and Dean, 2025) |
| CMPK2 | *C. trachomatis* | Modulation of immune response | (Olagoke, Chittaranjan and Dean, 2025) |
| CXCL10 | *C. trachomatis* | Leukocyte recruitment | (Olagoke, Chittaranjan and Dean, 2025) |
| CXCL2 | *C. trachomatis* | Leukocyte recruitment Modulation of inflammation | (Olagoke, Chittaranjan and Dean, 2025) |
| PTGS2 | *C. trachomatis* | Modulation of inflammation | (Olagoke, Chittaranjan and Dean, 2025) |
| IL1B | *C. trachomatis* | Modulation of inflammation Leukocyte recruitment | (Olagoke, Chittaranjan and Dean, 2025) |
| FOS | *C. trachomatis* | Stress response Modulation of inflammation | (Olagoke, Chittaranjan and Dean, 2025) |
| CXCL8 | *C. trachomatis* | Leukocyte recruitment | (Olagoke, Chittaranjan and Dean, 2025) |
| LGALS9 | *C. trachomatis* | Stress response Modulation of apoptosis Modulation of immune response | (Olagoke, Chittaranjan and Dean, 2025) |
| IL24 | *C. trachomatis* | Modulation of immune response Modulation of inflammation | (Olagoke, Chittaranjan and Dean, 2025) |
| CCL2 | *C. trachomatis* | Leukocyte recruitment | (Olagoke, Chittaranjan and Dean, 2025) |
| CSF2 | *C. trachomatis* | Leukocyte recruitment | (Olagoke, Chittaranjan and Dean, 2025) |
| PLSCR1 | *C. trachomatis* | Modulation of immune response | (Olagoke, Chittaranjan and Dean, 2025) |
| NCF2 | *C. trachomatis* | Modulation of oxidative stress | (Olagoke, Chittaranjan and Dean, 2025) |
| IRAK2 | *C. trachomatis* | Modulation of NF‑κB and MAPK signalling | (Olagoke, Chittaranjan and Dean, 2025) |
| S1P | *L. pneumophila* | Cell survival Trafficking  Modulation of autophagy | (Rolando *et al.*, 2016; Sahr *et al.*, 2017) |

**References supplementary material**

Andersson, A.-M. *et al.* (2016) “Autophagy induction targeting mTORC1 enhances *Mycobacterium tuberculosis* replication in HIV co-infected human macrophages,” *Scientific Reports*, 6(1), p. 28171. Available at: https://doi.org/10.1038/srep28171.

Berkova, N. *et al.* (2025) “Host DNA damage and cellular fate in bacterial infections, with a focus on *Staphylococcus aureus*,” *FEMS Microbiology Reviews*, 49. Available at: https://doi.org/10.1093/femsre/fuaf052.

Bose, D. *et al.* (2019) “Inhibition of TGF-β induced lipid droplets switches M2 macrophages to M1 phenotype,” *Toxicology in Vitro*, 58, pp. 207–214. Available at: https://doi.org/10.1016/j.tiv.2019.03.037.

Bravo-Santano, N. *et al.* (2018) “Intracellular *Staphylococcus aureus* Modulates Host Central Carbon Metabolism To Activate Autophagy,” *mSphere*, 3(4). Available at: https://doi.org/10.1128/mSphere.00374-18.

Bravo-Santano, N. *et al.* (2019) “Host-directed kinase inhibitors act as novel therapies against intracellular *Staphylococcus aureus*,” *Scientific Reports*, 9(1), p. 4876. Available at: https://doi.org/10.1038/s41598-019-41260-8.

Castañera, P. *et al.* (2026) “Host-directed microRNA-based intervention against intracellular *Staphylococcus aureus*: high-throughput screening identifies miR-4430, miR-147a, and miR-1249-5p as multifunctional antimicrobial candidates,” *Frontiers in Cellular and Infection Microbiology*, 16. Available at: https://doi.org/10.3389/fcimb.2026.1772100.

Cheng, C.Y. *et al.* (2017) “Host sirtuin 1 regulates mycobacterial immunopathogenesis and represents a therapeutic target against tuberculosis,” *Science Immunology*, 2(9). Available at: https://doi.org/10.1126/sciimmunol.aaj1789.

Demiroz, D. *et al.* (2021) “*Listeria monocytogenes* infection rewires host metabolism with regulatory input from type I interferons,” *PLOS Pathogens*, 17(7), p. e1009697. Available at: https://doi.org/10.1371/journal.ppat.1009697.

Eldridge, M.J.G. and Hamon, M.A. (2021) “Histone H3 deacetylation promotes host cell viability for efficient infection by *Listeria monocytogenes*,” *PLOS Pathogens*, 17(12), p. e1010173. Available at: https://doi.org/10.1371/journal.ppat.1010173.

Gutierrez, M.G. *et al.* (2004) “Autophagy Is a Defense Mechanism Inhibiting BCG and *Mycobacterium tuberculosis* Survival in Infected Macrophages,” *Cell*, 119(6), pp. 753–766. Available at: https://doi.org/10.1016/j.cell.2004.11.038.

Heim, C.E. *et al.* (2020) “Lactate production by *Staphylococcus aureus* biofilm inhibits HDAC11 to reprogramme the host immune response during persistent infection,” *Nature Microbiology*, 5(10), pp. 1271–1284. Available at: https://doi.org/10.1038/s41564-020-0756-3.

Howden, B.P. *et al.* (2023) “*Staphylococcus aureus* host interactions and adaptation,” *Nature Reviews Microbiology*, 21(6), pp. 380–395. Available at: https://doi.org/10.1038/s41579-023-00852-y.

Hresko, R.C. and Hruz, P.W. (2011) “HIV Protease Inhibitors Act as Competitive Inhibitors of the Cytoplasmic Glucose Binding Site of GLUTs with Differing Affinities for GLUT1 and GLUT4,” *PLoS ONE*, 6(9), p. e25237. Available at: https://doi.org/10.1371/journal.pone.0025237.

Idrovo, J.P. *et al.* (2016) “Inhibition of lipogenesis reduces inflammation and organ injury in sepsis,” *Journal of Surgical Research*, 200(1), pp. 242–249. Available at: https://doi.org/10.1016/j.jss.2015.06.059.

Jiang, J. *et al.* (2021) “Metabolomics Strategy Assisted by Transcriptomics Analysis to Identify Potential Biomarkers Associated with Tuberculosis,” *Infection and Drug Resistance*, Volume 14, pp. 4795–4807. Available at: https://doi.org/10.2147/IDR.S330493.

Juárez, E. *et al.* (2016) “Loperamide Restricts Intracellular Growth of *Mycobacterium tuberculosis* in Lung Macrophages,” *American Journal of Respiratory Cell and Molecular Biology*, 55(6), pp. 837–847. Available at: https://doi.org/10.1165/rcmb.2015-0383OC.

Kauppinen, A. *et al.* (2013) “Antagonistic crosstalk between NF-κB and SIRT1 in the regulation of inflammation and metabolic disorders,” *Cellular Signalling*, 25(10), pp. 1939–1948. Available at: https://doi.org/10.1016/j.cellsig.2013.06.007.

Kim, J.-S., Kim, Y.-R. and Yang, C.-S. (2020) “Host-Directed Therapy in Tuberculosis: Targeting Host Metabolism,” *Frontiers in Immunology*, 11. Available at: https://doi.org/10.3389/fimmu.2020.01790.

Modak, R. *et al.* (2014) “Epigenetic response in mice mastitis: Role of histone H3 acetylation and microRNA(s) in the regulation of host inflammatory gene expression during *Staphylococcus aureus* infection,” *Clinical Epigenetics*, 6(1), p. 12. Available at: https://doi.org/10.1186/1868-7083-6-12.

Moon, J.-S. *et al.* (2015) “UCP2-induced fatty acid synthase promotes NLRP3 inflammasome activation during sepsis,” *Journal of Clinical Investigation*, 125(2), pp. 665–680. Available at: https://doi.org/10.1172/JCI78253.

Nicolaou, G., Goodall, A.H. and Erridge, C. (2012) “Diverse Bacteria Promote Macrophage Foam Cell Formation Via Toll-Like Receptor-Dependent Lipid Body Biosynthesis,” *Journal of Atherosclerosis and Thrombosis*, 19(2), pp. 137–148. Available at: https://doi.org/10.5551/jat.10249.

Nicolas, A. *et al.* (2022) “Transcriptome Architecture of Osteoblastic Cells Infected With *Staphylococcus aureus* Reveals Strong Inflammatory Responses and Signatures of Metabolic and Epigenetic Dysregulation,” *Frontiers in Cellular and Infection Microbiology*, 12. Available at: https://doi.org/10.3389/fcimb.2022.854242.

Odçikin, E. *et al.* (2002) “Investigation of red blood cell carbonic anhidrase, glucose 6-phosphate dehydrogenase, hexokinase enzyme activities, and zinc concentration in patients with hyperthyroid diseases,” *Endocrine Research*, 28(1–2), pp. 61–68. Available at: https://doi.org/10.1081/ERC-120004538.

Olagoke, O., Chittaranjan, S. and Dean, D. (2025) “Transcriptional profiling of *Chlamydia trachomatis* and its host in an ex vivo endocervical primary cell culture system using dual RNA sequencing,” *Frontiers in Cellular and Infection Microbiology*, 15. Available at: https://doi.org/10.3389/fcimb.2025.1613922.

Palsson-McDermott, E.M. *et al.* (2015) “Pyruvate Kinase M2 Regulates Hif-1α Activity and IL-1β Induction and Is a Critical Determinant of the Warburg Effect in LPS-Activated Macrophages,” *Cell Metabolism*, 21(1), pp. 65–80. Available at: https://doi.org/10.1016/j.cmet.2014.12.005.

Pérez-Novo, C.A. and Bachert, C. (2015) “DNA methylation, bacteria and airway inflammation,” *Current Opinion in Allergy & Clinical Immunology*, 15(1), pp. 27–32. Available at: https://doi.org/10.1097/ACI.0000000000000130.

Rena, G., Hardie, D.G. and Pearson, E.R. (2017) “The mechanisms of action of metformin,” *Diabetologia*, 60(9), pp. 1577–1585. Available at: https://doi.org/10.1007/s00125-017-4342-z.

Rolando, M. *et al.* (2016) “*Legionella pneumophila* S1P-lyase targets host sphingolipid metabolism and restrains autophagy,” *Proceedings of the National Academy of Sciences*, 113(7), pp. 1901–1906. Available at: https://doi.org/10.1073/pnas.1522067113.

Russell, C.D. *et al.* (2025) “Genome-Scale Meta-analysis of Host Responses to *Staphylococcus aureus* Identifies Pathways for Host-Directed Therapeutic Targeting,” *The Journal of Infectious Diseases*, 232(2), pp. e290–e300. Available at: https://doi.org/10.1093/infdis/jiaf290.

Sahr, T. *et al.* (2017) “The *Legionella pneumophila* genome evolved to accommodate multiple regulatory mechanisms controlled by the CsrA-system,” *PLOS Genetics*, 13(2), p. e1006629. Available at: https://doi.org/10.1371/journal.pgen.1006629.

Singhal, A. *et al.* (2014) “Metformin as adjunct antituberculosis therapy,” *Science Translational Medicine*, 6(263). Available at: https://doi.org/10.1126/scitranslmed.3009885.

Song, Y. *et al.* (2018) “Inhibition of lactate dehydrogenase A suppresses inflammatory response in RAW 264.7 macrophages,” *Molecular Medicine Reports* [Preprint]. Available at: https://doi.org/10.3892/mmr.2018.9678.

Tannahill, G.M. *et al.* (2013) “Succinate is an inflammatory signal that induces IL-1β through HIF-1α,” *Nature*, 496(7444), pp. 238–242. Available at: https://doi.org/10.1038/nature11986.

Tataranni, T. *et al.* (2019) “Dichloroacetate Affects Mitochondrial Function and Stemness-Associated Properties in Pancreatic Cancer Cell Lines,” *Cells*, 8(5), p. 478. Available at: https://doi.org/10.3390/cells8050478.

Vachharajani, V.T. *et al.* (2016) “Sirtuins Link Inflammation and Metabolism,” *Journal of Immunology Research*, 2016, pp. 1–10. Available at: https://doi.org/10.1155/2016/8167273.

Yang, C.-S. *et al.* (2014) “The AMPK-PPARGC1A pathway is required for antimicrobial host defense through activation of autophagy,” *Autophagy*, 10(5), pp. 785–802. Available at: https://doi.org/10.4161/auto.28072.

Yu, J. *et al.* (2023) “Combining multi‐omics analysis to identify host‐targeted targets for the control of *Brucella* infection,” *Microbial Biotechnology*, 16(12), pp. 2345–2366. Available at: https://doi.org/10.1111/1751-7915.14307.

Zhong, W. *et al.* (2019) “Inhibition of glycolysis alleviates lipopolysaccharide‐induced acute lung injury in a mouse model,” *Journal of Cellular Physiology*, 234(4), pp. 4641–4654. Available at: https://doi.org/10.1002/jcp.27261.
